# Adaptive Transcriptome Profiling of Subterranean Zokor, *Myospalax baileyi*, to High- Altitude Stresses in Tibet

**DOI:** 10.1038/s41598-018-22483-7

**Published:** 2018-03-16

**Authors:** Zhenyuan Cai, Liuyang Wang, Xiaoying Song, Somnath Tagore, Xiangfeng Li, Huihua Wang, Jiarui Chen, Kexin Li, Zeev Frenkel, Dahai Gao, Milana Frenkel-Morgenstern, Tongzuo Zhang, Eviatar Nevo

**Affiliations:** 1 Key Laboratory of Adaptation and Evolution of Plateau Biota, Northwest Institute of Plateau Biology, Chinese Academy of Sciences, Xining, 810008 China; 2 University of Chinese Academy of Sciences, No.19A Yuquan Road, Beijing, 100049 China; 3 Department of Molecular Genetics and Microbiology, Duke University Medical Center, Durham, NC 27708 USA; 4 School of Life Sciences, Zhengzhou University, Zhengzhou, 450001 Henan China; 5 The Azrieli Faculty of Medicine, Bar-Ilan University, Safed, 13195 Israel; 6 Institute of Apicultural Research, Chinese Academy of Agricultural Sciences, Beijing, 100093 China; 7 The Institute of Evolution, University of Haifa, 199 Aba Khoushy Ave, Mount Carmel, Haifa 3498838 Israel; 8 Key Laboratory of Experimental Marine Biology, Institute of Oceanology, Chinese Academy of Sciences, Qingdao, 266071 Shandong China; 9 Qinghai Provincial Key Laboratory of Animal Ecological Genomics, Xining, 810008 China

## Abstract

Animals living at high altitudes have evolved distinct phenotypic and genotypic adaptations against stressful environments. We studied the adaptive patterns of altitudinal stresses on transcriptome turnover in subterranean plateau zokors (*Myospalax baileyi*) in the high-altitude Qinghai-Tibetan Plateau. Transcriptomes of zokors from three populations with distinct altitudes and ecologies (**L**ow: 2846 m, **M**iddle: 3282 m, **H**igh: 3,714 m) were sequenced and compared. Phylogenetic and principal component analyses classified them into three divergent altitudinal population clusters. Genetic polymorphisms showed that the population at **H**, approaching the uppermost species boundary, harbors the highest genetic polymorphism. Moreover, 1056 highly up-regulated UniGenes were identified from **M** to **H**. Gene ontologies reveal genes like *EPAS1* and *COX1* were overexpressed under hypoxia conditions. *EPAS1*, *EGLN1*, and *COX1* were convergent in high-altitude adaptation against stresses in other species. The fixation indices (*F*_*ST*_ and *G*_*ST*_)-based outlier analysis identified 191 and 211 genes, highly differentiated among **L**, **M**, and **H**. We observed adaptive transcriptome changes in *Myospalax baileyi*, across a few hundred meters, near the uppermost species boundary, regardless of their relatively stable underground burrows’ microclimate. The highly variant genes identified in *Myospalax* were involved in hypoxia tolerance, hypercapnia tolerance, ATP-pathway energetics, and temperature changes.

## Introduction

Zokors, genus *Myospalax*, are burrowing rodents that resemble mole rats, *Spalax*. In many subterranean mammals, like the *Myospalax baileyi* in Tibet, environmental stresses play a major role in their adaptive evolution^1^. Though they reside underground, Tibetan plateau zokors may have transcriptomic changes that might correlate with altitude. Notably, extinction ensues if organisms can’t adapt to the changing environmental stresses^2^. In addition, high-altitude stresses like solar radiation, hypoxia, hypercapnia, low temperature, and food shortage work in concert imposing interactive physiological challenges on these organisms. In regions such as the Tibetan plateau, solar radiation increases 8% ± 2% per 1000 m with elevation^3^, whereas temperature decreases by approximately 6 °C/km as the altitude increases^4,5^. The temperature and humidity within the burrow system of the plateau zokors are positively correlated with the air temperature and humidity above ground, despite that the burrow atmosphere is relatively stable^6^. Likewise, hypoxia in high altitudes coupled with the hypoxic subterranean environment reduces the oxygen supply to the respiratory tissues and cells, and influences the rate of metabolism^7–9^. Many animals adapt to such hypoxia by suppressing the rate of metabolism, or reducing oxygen demand^10^, including torpor or dormancy. However, homeothermic subterranean mammals which inhabit cold environments must sustain heat production to compensate for the cold stress. This results in producing sufficient energy to perform heavy daily digging activity, which may increase the metabolism rate and oxygen consumption^11^. Thus, deciphering the molecular genomic and transcriptomic mechanisms for adaptation of subterranean mammals to high altitude, such as on the Qinghai-Tibetan Plateau, will contribute to a better understanding of adaptive evolution.

The gas composition in underground burrows is affected by many factors including seasonal change, soil type, rain, depth of burrows, and metabolism of the animal^12–14^. Much rain occurs during summer on the Tibetan Plateau, thereby blocking the pores within the soil. Likewise, the gas permeability of the frozen soil in winter is also limited. The combination of all these factors limits the gas exchange between the underground burrow and atmospheric air. Furthermore, hypoxia increases with altitude and burrow depth^14,15^. Heavy soil on the Tibetan plateau significantly blocks air permeability^16^. Adapting to all of these severe stresses, organisms evolve diverse strategies including molecular genomic changes^17,18^ and alternative splicing^19^. Examples of such adaptations include deer mice in high altitudes that enhance their thermogenic capacity by oxidizing lipids as a primary metabolic fuel source^10^. Specific adaptive genes associated with altitudinal stresses have also been highlighted in humans^20–27^, dogs^28,29^, and leopards^30^. Most of the Qinghai-Tibetan Plateau animals studied live above-ground, but there is insufficient information on subterranean animals, on their altitudinal stress response despite their underground relative atmospheric shelter, primarily regarding humidity and temperature.

Plateau zokors (*Myospalax baileyi*) are blind subterranean mammals living from 2,600 m to 4,600 m^31^ on the Tibetan Plateau, spending nearly all their lives underground^32,33^. Plateau zokors burrow down to a depth of 70–250 cm underground, due to the frozen soil during the winter from November to March^34^. Similar to other subterranean mammals^35–37^, plateau zokors are also subjected to stresses such as hypoxia, *hypercapnia* and darkness, constant energy requirement during digging and exposure to pathogens, as in *Spalax*^38^. Noteworthy, the brain size of S*palax* increases with higher environmental stresses^39^. Likewise, the brain is the most sensitive organ to hypoxia. Thus, we have chosen to use brain tissue in our *Myospalax* experiments.

**Figure 1:**
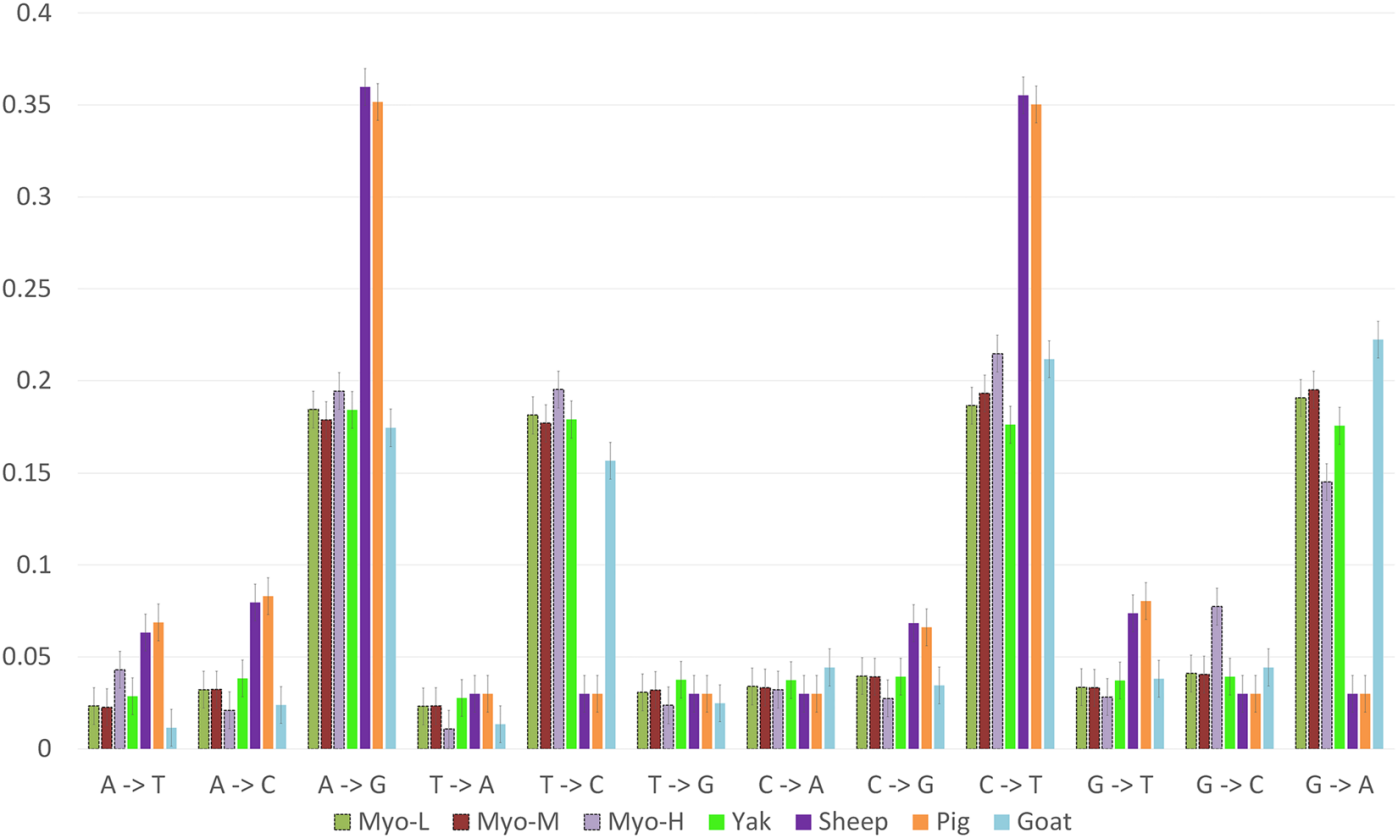
The comparative SNPs analysis of zokors with respect to the SNPs frequencies in Tibetan yaks (~3500 m), sheep (~2300–3500 m), goats (~3000 m), and pigs (~3000–4000 m). We observed that in zokors, the average SNP was higher at the **H** altitudes for G −> A, A −> G, T −> C, C −> T, G −> C, comparing the data with altitudes.

In this study, we identified 1,056 differentially expressed genes whose expression was upregulated upward across the short distance of 432 m from **M** to **H**. We hypothesize that this increase in upregulation upward resists some of the high Tibetan altitudinal stresses (solar radiation, cold temperature, hypoxia, and food scarcity). This is especially critical in the upper level of 4,000+ m approaching the species uppermost boundary in Tibet. Moreover, gene ontology analyses revealed that putatively selected genes, like *EPAS1* and *COX1*, were overexpressed under hypoxia conditions^24^. We used the comparative SNPs frequencies analysis with other Tibetan animals and meta-analysis to verify our findings at the different altitudes for zokors. Figure 1 represents a comparative SNPs analysis of zokors with respect to the SNPs frequencies in the Tibetan yaks (~3500 m), sheep (~2300–3500 m), goats (~3000 m), and pigs (~3000–4000 m). We observe that in zokors, the average SNP was higher at the **H** altitude for G −> A, A −> G, T −> C, C −> T, and G −> C, comparing the data with altitudes. Moreover, we showed that natural selection, which was substantiated statistically, has adaptively molded zokor’s transcriptome between distinctly divergent populations of the same subterranean rodent species, *Myospalax baileyi*, and across a few hundred meters, to adaptively and effectively cope with Qinghai-Tibetan Plateau altitudinal stresses.

## Results

### *De novo* transcriptome assembly and annotation

**Figure 2:**
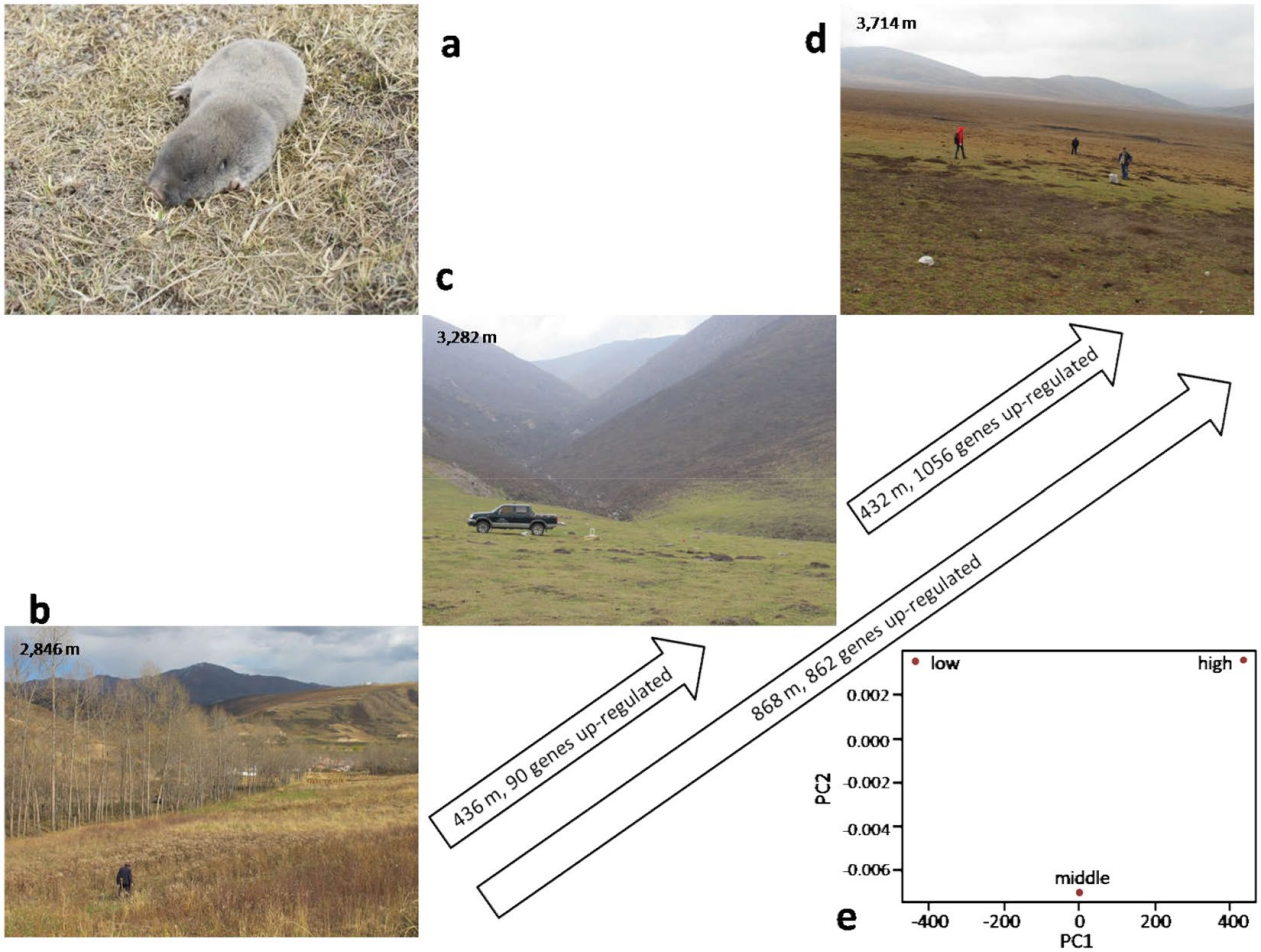
Study animal and three ecologically divergent sampling sites, representing altitudinal populations with increasing environmental stresses. (**a**) Plateau zokor, *Myospalax baileyi*; (**b**) Low sampling site, **L**, 2846 m, grassland; (**c**) Middle sampling site, **M**, 3282 m, alpine steppe. (**d**) High sampling site, **H**, 3714 m, extreme alpine steppe. (**e**) PCA of the multiple environmental stresses on the three altitudinal populations.

Three plateau zokors populations (Fig. 2a) from each altitude: low (**L**, 2,846 m), middle (**M**, 3,282 m), and high (**H**, 3,714 m), in the Tibetan Plateau (Fig. 2b–d, Table S1) were collected for our transcriptome study and analyzed by high-throughput RNA sequencing. The three sample sites correspond to three divergent populations varying in environmental temperature, UV, oxygen, and food resources (Fig. 2e, Table S2). All the animals were adult males, and the tissues used in this study were from whole brains, due to the high consumption of, and sensitivity to, oxygen in the brain. The brain was chosen as the study organ as it is known to be highly sensitive to hypoxia. After removing all the adaptors, the low-quality reads as well as those with more than 5% N were removed. The total number of high-quality reads for each individual ranged from 40.7 to 49.7 M, totaling 362.6 M reads for all nine sample animals (Tables S1–S3). For the paired-end high-quality reads, we used *de novo* RNA-Seq assembly and performed further analysis using Trinity and EdgeR, with the default parameters and the minimum *kmer* coverage of 2^40^. The reads were assembled into 233,547 UniGenes that have been subjected to further clustering forming longer assembled sequences without *N*s with the *N*50 length of 2,928 bp and mean length of 1,381 bp (Table S4). The length distributions of all the UniGenes, blasted CDS, and predicted CDS are shown in Figs. S1, S2, and S3, respectively. In total, 165,527 UniGenes were annotated for the available references in the *Nr* (Fig. S4), *Nt*, Swissprot, COG (Fig. S5), KEGG (Fig. S6), and GO databases (Fig. S7). The numbers of unique hits for the databases were 111,461, 163,747, 106,727, 43,558, and 85,714, 89,955, respectively (Fig. S6). As stresses like cold temperature, low-food resources, high UV, high hypoxia, and hypercapnia are extreme at the high altitudes, many genes are expected to be involved in the adaptation, especially near the highest species border (4,600 m).

### SNP calling and cluster analysis

**Figure 3:**
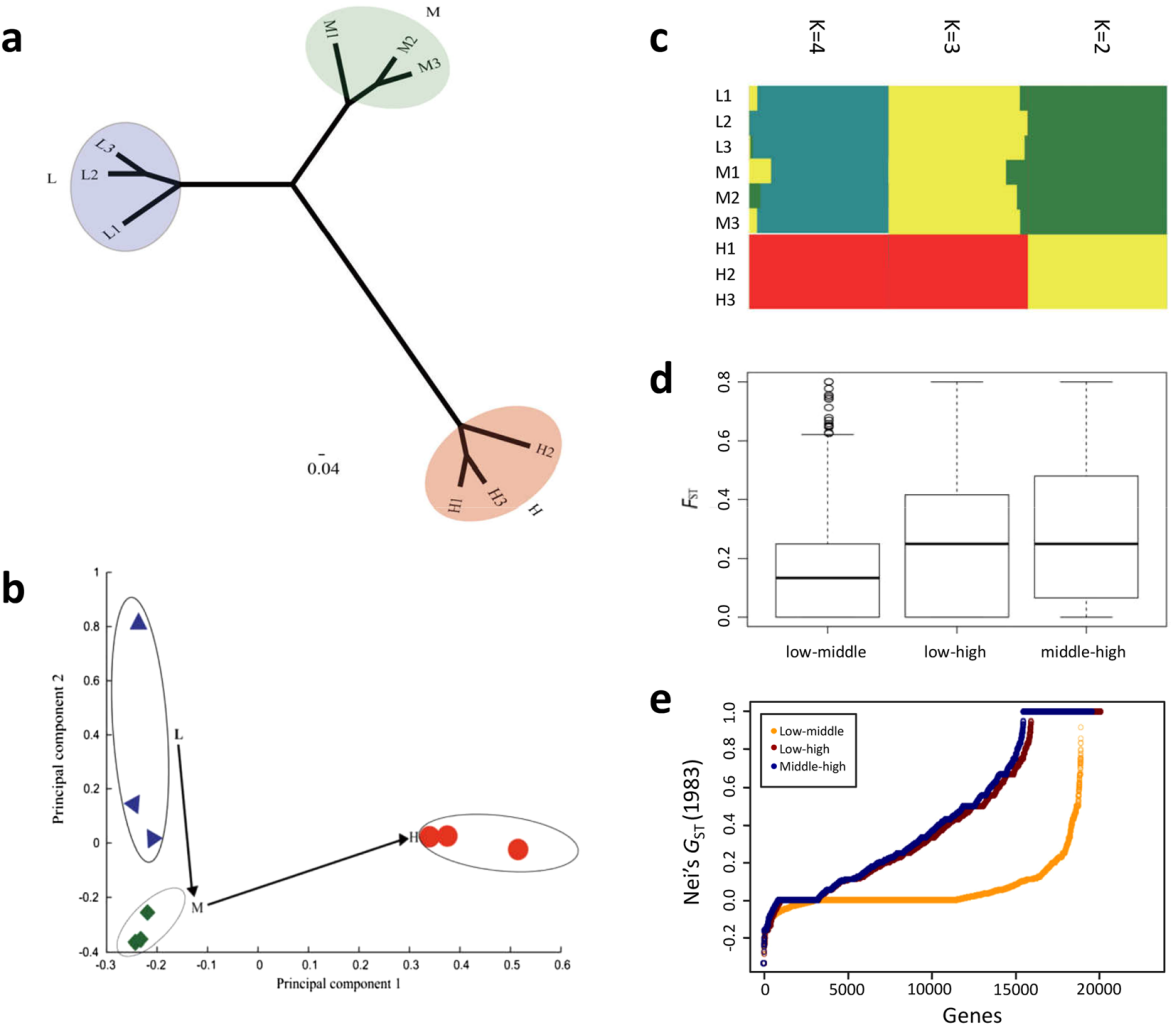
Distinct genetic differentiation among ecologically divergent zokor populations at three altitudes. (**a**) Neighbor joining tree. **L1**, **L2**, and **L3** are zokors from low-altitude populations (2,846 m); **M1**, **M2**, and **M3** are zokors from middle altitude populations (3,282 m); and **H1**, **H2**, and **H3** are zokors from high-altitude populations (3,714 m). (**b**) Principal component analysis (PCA) of the three altitudinal populations. Samples from low altitudes (**L**) are marked in blue triangles, from middle altitudes (**M**) are marked in green squares, and samples from high altitudes (**H**) are marked in red circles. Note the dramatic genetic divergence of **H** from both **L** and **M**. (**c**) Altitudinal population structure when k = 2, 3 (green, yellow, and red) and K = 4 (green, yellow, red, and blue). Here, K is defined as the number of (ancestral) populations. The higher the K, the more fine-structured (tuned) is the population subdivided into more units. (**d**) Measurement of *F*_ST_ for all genes among the three populations, indicating high-genetic differentiation between low, middle, and high altitudes. It shows that both **L-H** (0.428) and **M-H** (0.452) have higher FST than **L-M** (0.063). (**e**) *G*_ST_ differentiation of the three altitudinal populations. Note the difference between the low and other two altitudinal populations.

The SNP number for the nine individuals ranged from 12,200 to 26,559 (Table S5), and the transversion rate ranged from 2.6 to 2.8. The SNP density for **L**, **M**, and **H** populations are 45.07 ± 2.45/Mb, 42.29 ± 5.62/Mb, and 78.48 ± 2.29/Mb, respectively. The difference in SNP density in the **L** *vs*. **M** populations was not significant (two-sample pooled t-test with equal variances, *P* = 0.455). However, the SNPs density was significantly different for the comparisons of **L** *vs*. **H** (*P* = 0.005) and **M** *vs*. **H** populations (two-sample pooled t-test with equal variances, *P* = 0.004) (Table S6). Three distinct altitudinal population clusters were identified by both the Neighbor-Joining (NJ) method (Fig. 3a) and Principal Component Analysis (PCA) (Fig. 3b) based on 14,200 SNPs, covering reads in all nine individuals spread across the transcriptome. In PCA, the first component could clearly differentiate the **H** from the **L** or **M**, and the **L** population was differentiated from the **M** population by the second component. The first and second components explained 41.1% and 10.36% of the SNP variances, respectively (Fig. 3b). All together, these results indicate that the low (**L**) and middle (**M**) altitudinal populations are similar but they were very different from the high-altitude population **H** (Fig. 3c) due to its exposure to the stressful climax uppermost environment. To confirm these findings, we identified the SNP frequencies reported in other Tibetan animals at high altitudes, e.g., Tibetan yaks, goats, sheep, and pigs^41^. Remarkably, other animals showed similar alterations in the SNPs frequencies as in the zokors, resulting in similar patterning. Particularly, the **H** altitude frequencies were significantly different from other altitudes for zokors, (FDR < 1%), and produced the lower variants in G−> A, and the higher variants in G−> C (Fig. 1). In general, the structure showed by PCA could be the result of several factors including altitudinal stresses, population sub-structure, and so on. However, in this study, the GO enrichment of differentiated genes between different populations were mainly related to altitude; meanwhile, the mitochondrial study by Tang *et al*.^31^ showed there was no sub-structures among the three populations that were caused by geography^31^.

### Genetic diversity

The number of polymorphic loci of the three populations has been investigated. Notably, the high-altitude population, **H**, displays the largest proportion of polymorphic loci. The genetic diversity of the three populations was estimated by both θ and л. The θ value was 3.88 × 10^−6^, 3.26 × 10^−6^, and 5.56 × 10^−6^ for **L**, **M** and **H**, while the л value was 3.90 × 10^−6^, 3.39 × 10^−6^, and 5.84 × 10^−6^, respectively (Table S7). The **H** population showed significantly higher genetic diversity than both **L** and **M**. *F*_ST_ statistics showed the genetic distance of **L** and **M** is 0.083, while the *F*_ST_ between **L** and **H**, as well as **M** and **H**, is 0.428 and 0.452, respectively. Moreover, there were 1,462, 2,099, and 8,478 SNPs unique to **L**, **M**, and **H** populations, respectively (Fig. S8a). The shared SNPs between **L** and **H** and between **M** and **H** populations were only 386 and 502, respectively, while **L** and **M** populations share 1,751 common SNPs (Fig. S8a). The differences of SNPs within genes are significantly higher (*P* < 0.0001) at **H**, but not at **L** and **M**. The average SNP numbers within genes, and across the whole data set of the **H** group are 1.18 and 1.5, respectively (Fig. S8b), whereas the numbers were significantly lower within SNPs among genes in **L** (t-test, *P* < 0.00001). The proportion of SNPs within genes unique to the **L**, **M**, and **H** were 0.004, 0.003, and 0.085, and SNPs across the whole data set were 0.027, 0.018, and 0.517, respectively. The proportion of unique SNPs in the **H** population was significantly higher than that in **L** and **M** (t-test, P < 0.00001). The difference in proportion between SNPs across the whole data set and SNPs within genes in **H** was significant (t-test, *P* < 0.00001) (Fig. S8b). Remarkably, the most dramatic increase in the SNP number occurs from **M** to **H**, across only 432 m. Similarly, the number of synonymous and non-synonymous SNPs, SNPs in UTRs, and CDS show the same trend, i.e., increases with the rise in altitude (Fig. S8c,d). The highest non-synonymous SNPs causing changes in amino acids occur in the most stressful site in **H**, at 3714 m, approaching the uppermost species altitudinal boundary (Fig. S8d). SNPs in 3′UTR show higher density than in either 5′UTRs or CDS (Fig. S8c). These results are due to the lower stress between the two populations, **L** and **M**. By contrast, the greatest differences are in the last upper 432 m between **M** and **H**, where environmental stresses that climax dramatically (Fig. 2d).

### The differential gene expression and GO enrichment analyses

Genes and transcripts from the three ecologically divergent altitudinal populations were quantified by Reads per Kilobase of transcript per Million mapped reads (RPKM). Comparisons of gene expression levels of **L** vs. **M**, **M** vs. **H**, and **L** vs. **H** show that 99, 1,056, and 862 UniGenes were significantly up-regulated, while 150, 488, and 357 UniGenes were down-regulated, respectively (Fig. S9a). A comparison among **L**, **M**, and **H** populations showed only 50 UniGenes significantly up-regulated and 8 genes down-regulated. The gene expression profiles for all transcripts were displayed in nine clusters in Fig. S9b. Hierarchical clustering analyses of the differentially expressed genes (DEGs) [ANOVA with FDR correction, *P* < 0.05, |log_2_ (Fold Change) |>1] of animals from **L** vs. **M**, **M** vs. **H**, and **L** vs. **H** pairs (Fig. S10a–c) show expression profiles varied significantly in response to different altitude stresses. In the expression pattern of **L** vs. **H** and **M** vs. **H**, individuals could be separated into two altitudinal pairs. However, an outlier (*L*1) was found in the **L** vs. **M** pair. DEGs from each pairwise altitudinal group were shown by means of volcano plots (Fig. S10d–f), where the difference between **M** and **H** was the largest^42^. The data points in blue and red, representing the genes that met the applied cut-offs, were used to identify biologically (magnitude) and statistically significant changes for each pairwise comparison. Finally, the Gene Ontology (GO) analysis revealed that the potential adaptive genes were overexpressed in hypoxia and blood pressure regulation. Remarkably, *EPAS1*, *EGLN1*, and *COX1* were identified with specific signature, which correlated with high-altitude convergent adaptation in other species. The GO enrichment was performed on the DEGs in **L** vs. **M**, **M** vs. **H**, and **L** vs. **H**. The functional enrichment of DEGs between **L** and **H** revealed that up-regulated genes were mainly related to energetics, neurogenetics, and nutrition (Fig. S12), which cope adaptively with the major stresses increasing upward, but down-regulated genes were also enriched in GO terms related to energy (Fig. S12).

### Natural selection

**Figure 4:**
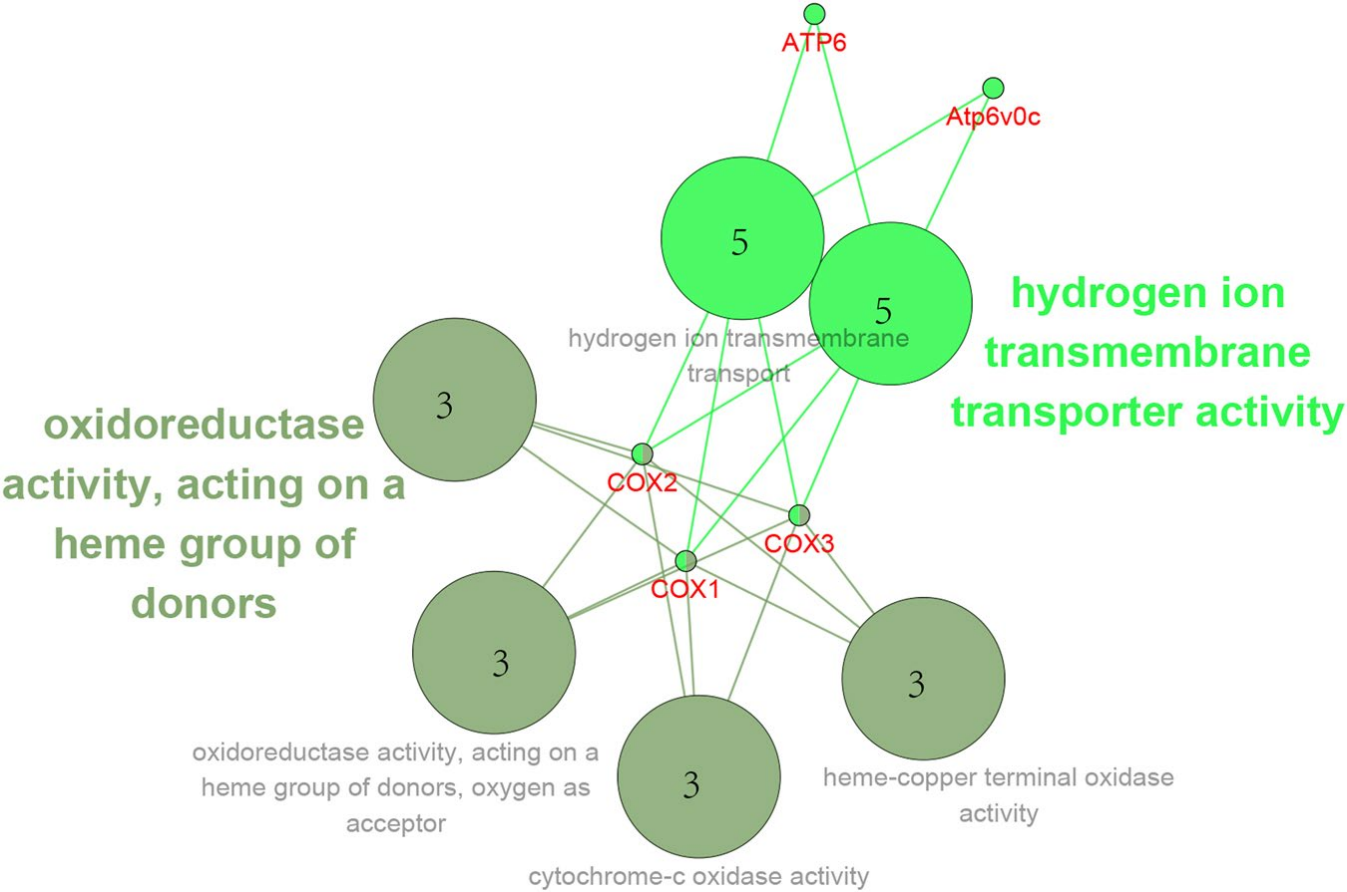
GO enrichment of positively selected genes of the high-altitude population. GO:0004129: Cytochrome-c oxidase activity; GO:0015002: Heme-copper terminal oxidase activity; GO:0015078: Hydrogen ion transmembrane transporter activity; GO:0016675: Oxidoreductase activity, acting on a heme group of donors; GO:0016676: Oxidoreductase activity, acting on a heme group of donors, oxygen as acceptor; GO:1902600: Hydrogen ion transmembrane transport.

The *F*_ST_ and *G*_ST_ parameters display population differentiation. *F*_ST_ and *G*_ST_ outlier genetic analyses showed that there were 191 and 211 genes (Supplementary Data) that were highly differentiated between **L**, **M**, and **H** with 149 shared genes. Notably, the genes of *APOPT1* and *COX1* showed large divergences in both **M** vs. **H**, and **L** vs. **H** pairs. The differences between **L** and **M** were the smallest, while the differences between **L** and **H**, and **M** and **H** were distinctly larger (Fig. 3d,e). The Tajima’s neutrality D test^43^ was performed and the distribution of D showed that animals from **H** have the largest proportion of SNPs with D > 1.5 (Fig. S13a) but have the smallest proportion of SNPs with D <−1 (Fig. S13b). Thus, for the **H** population, we performed a locus-specific analysis to identify candidate transcripts under selection. We defined candidate selected transcripts as those with a Locus-Specific Branch Length (LSBL) value higher than 0.5. Within the **H** group, 3,379 genes were considered putative signatures of selection. Remarkably, there were 1,039 genes with LSBL value equal to 1, which harbored some distinctive mutations. Some genes with large LSBL values, like *COX1* (LSBL_(*H*)_ = 1), *EPAS1*(LSBL_(*H*)_ = 0.75), and *EGLN1*(LSBL_(*H*)_ = 0.5), were identified with strong selection signature in **H**. Interestingly, regarding adaptive hypoxia tolerance, we found GO terms mainly involved in heme vascular endothelial (heme-copper***)*** terminal oxidase activity, oxidoreductase activity, acting on a heme group of donors and vascular growth as well as development (vascular endothelial growth factor signaling pathway, regulation of blood vessel endothelial cell migration, negative regulation of blood pressure) (Fig. 4), both of which play important roles in hypoxia adaptation. Finally, the sequences of two genes, *EPAS1* and *EGLN1* of *M*. *baileyi*, together with the sequences of another 25 species were multi-aligned. Interestingly, only one amino acid was found to be unique to *Myospalax* within the gene of *EGLN1* (**H** > **L**, 174) (Fig. S14a) and *EPAS1* (**T** > **S**, 472 and **L** > **P**, 576) (Fig. S14b,c).

### Approximate Bayesian Computation

**Figure 5:**
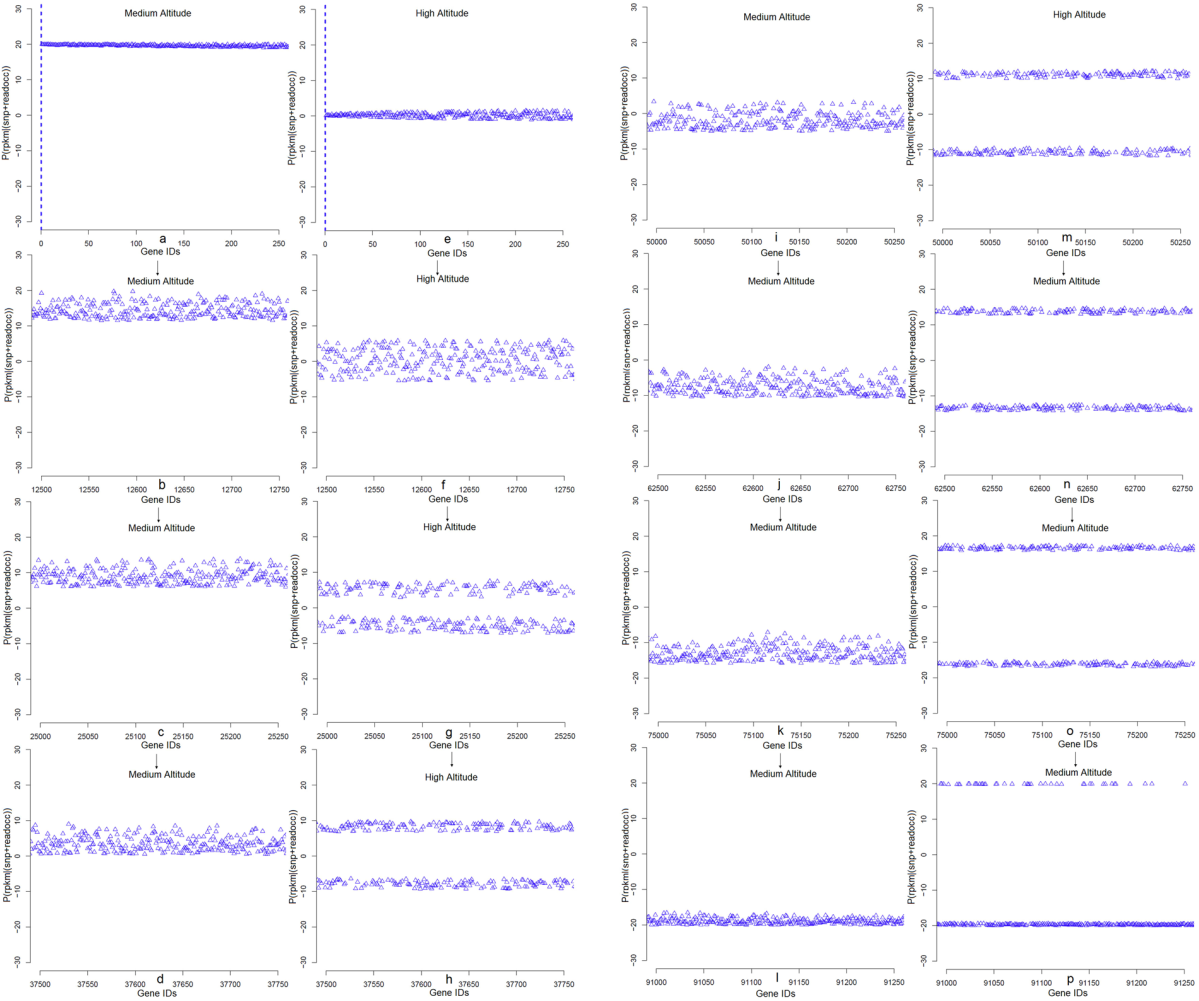
Approximate Bayesian Computation model shows that there is a clear bifurcation in SNP density in the highest altitude, **H**, where the cluster-mean increases. The cluster-mean for SNP density in the middle altitude did not show a significant increase (**a–p**).

The Approximate Bayesian computation (ABC) algorithm has been widely used in the scientific community to analyze population demography, growth rate, and time of divergence in the last decade^44^. ABC-based methods approximate the likelihood function by simulations, the outcomes of which are compared with the observed data. With the ABC rejection algorithm, a set of parameter points is first sampled from the prior distribution. The ABC model^44^ revealed that the gene expression level difference occurs due to genetic components (SNP mutation rate, read occurrence, and RPKM fluctuation), as environmental components were “fixed” during our computational implementation. Therefore, we claim, although we did not perform the common garden experiment, that gene expression level differences were strongly affected by genetic background and natural selection. The ABC model substantiates the conclusion that SNP density increases at the 432 m between the **M** population at 3,282 m and the **H** population at 3,714 m. To conclude, we showed that in *Myospalax* the gene expression level differences involve both *genetic* and *environmental* (plasticity) components. To separate the genetic and environmental (plasticity) effects on the transcriptome, the ABC model was implemented to show that there is a clear bifurcation in SNP density in the highest altitude, where the cluster-mean increases. The cluster-mean for SNP density in the middle altitude has not showed a significant increase (Fig. 5a–p). Next, we performed a comparison of SNP statistics for plateau zokor with respect to the Tibetan yaks, sheep, goats, and pigs SNPs densities. This analysis identified that the average SNP was higher in **H** for G 1> A, A −> G, T −> C, C −> T and G −> C. Comparing the data with other organisms: G −> A, A −> G, T −> C, C −> T is higher in yaks; A −> G, C −> T is higher in sheep; A −> G, T −> C, C −> T, G −> A is higher in goats, and A −> G, C −> T is higher in pigs (Fig. 1)^41,45^. Furthermore, the annotation of the plateau zokor genes has revealed a unique association with the various molecular pathways like cytokine signaling, angiogenesis, *CCKR* signaling, integrin signaling, *TGF-beta*, *PDGF* signaling, and heterotrimeric G-protein signaling (Fig. S15). In addition, the genes in *Myospalax* were annotated and their associations with environmental stresses like hypoxia tolerance, hypercapnia tolerance, ATP-pathway energetics, and temperature have been identified (Tables S9–S12). To conclude, first, we observed that expression profiles are indeed varying significantly from (**L + M**) to **H**, *i*.*e*., towards the higher stresses; second, the ABC model clearly indicates that there is a certain genetic component that leads to the changes in the SNP density at the highest altitude.

### The meta-analysis used to compare different studies in Tibetan animals

Meta-analysis was done to improve the sample selection and highlight the data divergence and convergence^41,46^. The analysis included different species from each **L, M**, and **H** sites to support the genetic diversity and selection findings (Fig. 1). Figure 1 represents a comparative SNPs analysis of zokors with respect to the SNPs frequencies in the Tibetan yaks (~3500 m), sheep (~2300–3500 m), goats (~3000 m), and pigs (~3000–4000 m). We observe that in zokors, the average SNP was higher at the **H** altitudes for G −> A, A −> G, T −> C, C −> T, G −> C, comparing the data with altitudes. Moreover, we made a randomization of the samples based on the SNP density with respect to altitude^46^. We used the Hedges’ g value as a measure to eliminate the scale differences in different studies. We used the random meta-regression model to examine the impact of moderator variables on every study effect size, and the standard normalization for all the studies^46^. For our nine samples considered (three at each location), we randomized them into 27 studies (nine for each set). We used the SNP data for plateau zokor and calculated the p-values for a 95% confidence interval. The main reason for the randomization was a way to test our hypothesis: whether the p-values would improve for SNPs from **M** to **H** altitude. Thus, Tables S13–S14 show that at the 95% confidence interval, the observed p-values improved significantly for most of the studies, from **M** to **H** altitudes^41,45^. Thus, taken together with the previously made comparison of SNPs of zokors with other organisms, we identified the same alterations in the SNPs frequency in other studies on Tibetan animals at similar altitudes, as we have observed in zokors.

## Discussion

### Overall summary of results

In this study, the samples of plateau zokors were collected from different altitudes (2846-3714 m) to analyze the effect of the altitude on their transcriptome and SNPs density property. The relative small number of samples, three in each of the three populations (**L**, **M**, and **H**), have been compensated by the entire transcriptome analysis, also using a comparative and meta-analysis with other Tibetan animals^42^. The *de novo* assembly of RNA sequencing data was used to identify that the gene expression showed unique gender, and spatio-temporal and tissue-specific characteristics, as shown also in other organisms^47–50^. To eliminate abiotic and artificial effects caused by gender and tissue differences, only males were collected, and brains were harvested for transcriptome analysis, since the brain tissue is remarkably sensitive to oxygen levels and requires high oxygen for its function. All the animals were above 230 g (Table S1) and therefore considered mature adults^51^. The main differences between the three sample sites were altitudes and divergent environmental stresses (Fig. 2b–e). Furthermore, the transcriptomic changes were correlated with energetics, neurogenetics, and nutrition (Fig. S12). Using the PCA and NJ clustering analyses (Fig. 3a,b), it was found that animals from the same altitudinal population are clustered into the same group, and the three ecologically divergent populations are distinctly separated (Fig. 3a–e). Each population was ecologically divergent from the other two populations (Fig. 2e). The genetic distance between **L** and **M** clusters was shorter than the distance between **M** and **H**, although both differences represent the same altitudinal distance of about 400 m. This suggests that the uppermost 432 m, an astoundingly short distance, which is between 3,282 m and ∼3,714 m, holds a threshold for plateau zokors’ survival, where thousands of the UniGenes’ expressions show changes approaching the species uppermost boundary.

### Mutations across the three altitudes

The **L** and **M** populations show close relationships, but both were comparatively distant from the **H** population, as evidenced by the NJ clustering, PCA, *F*_ST_, and *G*_ST_ analyses (Fig. 3a–e). This result may be due to the drastic environmental stresses as altitude increases from **M** to **H**, at a very short distance of 432 m, near the uppermost border of the species range^31^ in the Tibetan Plateau. Although the distance from **L** to **H** is relatively short (862 m), environmental stress appears to play a key role in population differentiation, particularly between **M** (3,282 m) and **H** (3,714 m), approaching the biological threshold of the plateau zokors’ survival threshold. Mutations occurring in coding regions, especially those that are non-synonymous in nature, alter amino acids and hence influence the function or activity of proteins. This leads to the adaptations described as resisting multiple environmental stresses. In the present study, 643 non-synonymous SNPs were found in **H** as compared to 284 found in **L** and **323** found in **M**. As the altitude rises, especially from **M** to **H**, the environmental stresses are significantly accentuated, and genetic polymorphism dramatically increases. The association between genetic polymorphism and environmental stress was shown at local, regional, and global scales by allozyme, and DNA markers^39^, and at the SNP level^52^. This is most likely due to the increasing innovative adaptations and recombination to cope with climaxing environmental stresses. Particularly, it is the higher genetic polymorphism that enhances fitness and facilitates adaptations to the more severe stresses at a higher altitude^2^. Moreover, SNPs located in 3′ and 5′ UTR are important because they affect miRNA binding^53,54^. In this study, we found more SNPs in UTRs of the **H** population leading to high efficiency of gene expression and associated with the highest stress site in our samples (close to the upper altitude of zokors in Tibetan Plateau at 4,600 m), which is the uppermost boundary of the species *Myospalax baileyi*.

The SNPs shared by **M** and **H**, and **L** and **H** populations are only 2.46% and 4.68%, respectively. However, the shared SNPs between **L** and **M** populations are substantial: 44.53% for **M** and 47.54% for the **L** population. This suggests similar stresses between **L** and **M** but different due to severe stresses at high altitudes. This is quite remarkable as **H** is only 432 m above **M**. This 432 m inflicts higher stresses near the upper species range hence generating dramatic transcriptome responses (Figs. S7, S8, S13). The polymorphic loci, θ and л values, showed that the high-altitude population harbors the highest genetic diversity, which makes **H** the most stressful population out of the three tested and forces **H** animals to cope with more severe multiple environmental stresses^2,55^. Moreover, the hierarchical NJ clustering analysis (Fig. S10a–c) revealed that the pairwise altitudinal populations’ comparison has similar expression patterns, suggesting that the adaptation of animals to environmental stress involves gene expression level alterations^56^. The gene expression study also clearly showed three altitudinal populations, although **L1** was not clustered closely to **L2** and **L3**. The volcano plot (Fig. S10) significantly shows that there were more differentially expressed genes in **M** vs. **H** pairs, suggesting that the stress is more drastic in the uppermost 432 m than in the lower part of the altitudinal transect that of **L** vs. **M** pairs, indicating that there is some stress threshold in the **H** altitude for zokors’ survival. Food resources are scarce at higher compared to lower altitudes. Tolerance to poisons, such as alkaloid, phytoalexin, and even herbicides (Fig. S12), may help zokors enlarge their food spectrum^57–59^ in order to cope with the food scarcity at high altitudes. In addition, animals must also enlarge their home territory in order to reach as much food as possible. More developed neurogenetic systems improve magnetic navigations of animals to build intact maps of the burrow system^60^, which is displayed by GO categories related to neurogenetics (Fig. S12). *Spalax* brain size increases towards the Negev desert^39^. GO term of up-regulated genes showed antioxidant activity enrichment (Fig. S12) that protects tissues from hypoxic damage. This is displayed by the function of the positively selected gene of *APOPT1*. The zokor adaptive transcriptomic turnover from **M** to **H**, is presumably the strongest intraspecific response described to date in resisting multiple high-altitude Tibetan environmental stresses. This is especially interesting as zokors are partly protected in their underground burrows from aboveground climatic stresses.

### Natural selection on altitudinal populations

The allele number of SNPs across the whole data set is significantly higher in **H** than in **L** and **M** populations (Fig. S8b), highlighting the positive association between environmental stress and genetic polymorphism^55^. In addition, the number of SNP alleles within genes is lower at **H**, most likely because of the higher stresses. Tajima’s *D* analysis suggests that selection for more genetic polymorphism increases with altitude, and selection is the strongest at the highest altitude (Fig. S13). Locus-specific analyses identified that some genes (including *COX1*, *EPAS1*, and *EGLN1*) were strongly selected and harbored many unique mutations. These three genes were shown to be associated with high-altitude evolutionary convergent adaptation in many other species^21,61^. *APOPT1* plays an important role in the regulation of apoptosis, promoting programmed cell death^62^, which is cardinal in coping with hypoxia and avoiding cancer. In the present study, the *APOPT1* gene showed a large differentiation at **H**, most probably due to the upward increasing hypoxic stress at the higher altitude, thus decreasing the risk of hypoxia-induced damage as well as the increasing potential of cancer. Oxidative/reductive stresses occur at high altitudes where there is lower oxygen pressure^63^. *APOPT1* could suppress the level of reactive oxygen species^64^ to protect tissues from damage. The *COX1* gene is another outlier gene in both genomic variants and gene expression analysis, and is known to be associated with hypoxia adaptation^61,65^. *EPAS1* and *EGLN1* have also been reported as playing a significant role in the adaptation to Tibetan Plateau stresses^20–23,28,66^. Mutations unique to plateau zokors may also facilitate the adaptation to high-altitude Qinghai-Tibetan Plateau stresses, but further functional verification should be done to substantiate this in plateau zokors, and show experimentally that the **H** population is better in resisting hypoxia than other populations.

### Conclusions and Prospects

Stressful environments, such as the Tibetan Plateau, are excellent biological- evolutionary laboratories to decipher transcriptome evolution across very short distances. This is especially true if tests are conducted intra-specifically in populations at their extreme species ecological distribution ranges at higher altitudes. Our results in plateau zokors, *M*. *baileyi*, at three altitudinal populations in the Tibetan Plateau, 2,846 m (**L**), 3,282 m (**M**), and 3,714 m (**H**) demonstrated interesting transcriptome evolutionary adaptations. These adaptations positively correlate with altitude at a short distance of 868 m, but primarily at an even shorter distance of 432 m at the higher altitude, approaching the uppermost boundary of the species survival. Adaptive complexes generated by natural selection, as shown by the Tajima’s *D* test, involved SNP genetic polymorphisms, and GO elements including hypoxia, energetics, neurogenetics and nutrition, which correlated with the following environmental stresses: low-oxygen tension, cold temperature, and drought as well as food shortage. This is highlighted by stress genes displaying an increase in SNP density at the higher altitude, near ecological extremes of the species boundary. Therefore, our results uncovered the adaptive evolution of the subterranean zokor because of the highly stressful environment at different high altitudes in the Tibet Mountains. What is next? Epigenetic studies, changes in the methylation, microRNAs, and other regulators may highlight the regulatory mechanisms evolved at the high-altitude adaptation. The analysis of the noncoding regulatory elements and extended experimental lab work will probably identify the physiological increase in hypoxia resistance from **L** to **H** altitudes.

## Materials and Methods

All the operations on plateau zokors were approved by the Qinghai Forestry Bureau, and all animals were treated in accordance with Animal Care and Use Committee Rules of Northwest Institute of Plateau Biology, Chinese Academy of Sciences. Transcriptome sequencing was performed on animals from three altitudinally distinct populations of the Tibetan Plateau. Genetic divergence was estimated by the Neighbor-Joining (NJ) cluster method using TreeBeST v1.9.2, and by the principal component analysis (PCA) based on the SNPs across the transcriptome. The permutation test was conducted for PCA analysis to ecologically differentiate the **H** from **L** and **M**, at the p-value of 0.012, while the p-value for **L** and **M** was 0.2. Genetic polymorphism among the three altitudinal divergent populations was compared using SNP density. GO enrichment was performed on DEGs (differentially expressed genes) that were identified among the three altitudinal populations. Natural selection was detected by *F*_ST_ and *G*_ST_ -based fixation indices and Tajima’s *D* test. Finally, using the ABC algorithm, we used the SNP density at **L**, **M** and **H**, to understand whether there is any dramatic change in their measurement across altitudes. In this method of model-based statistical inference, a likelihood function expresses the probability of SNP density data under the Null-hypothesis, stating that there is no dramatic increase in SNP density with respect to altitude. The outcome of our ABC rejection algorithm clearly showed that SNP polymorphism density with increasing altitude includes all the genetic components. The individual ancestry was estimated by FRAPPE v 1.1. Genetic polymorphism among the three altitudes was compared using SNP density. The GO enrichment analysis was performed on DEGs that were identified among the three altitudinal populations by Database for Annotation, Visualization and Integrated Discovery (DAVID), a knowledge base of functional annotation tools.

## Electronic supplementary material

Supplementary Information
