# Supplementary material for: Adaptive Transcriptome Profiling of Subterranean Zokor, Myospalax baileyi, to High- Altitude Stresses in Tibet
Source: Sci Rep. 2018 Mar 16;8:4671. doi: 10.1038/s41598-018-22483-7 (PMC5856782; doi:10.1038/s41598-018-22483-7)
Supplement: Supplementary file 1 — Supplementary Information [file 41598_2018_22483_MOESM1_ESM.doc]

**Supplementary Information**

**Adaptive Transcriptome Profiling of Subterranean Zokor, *Myospalax baileyi*, to High Altitude Stresses in Tibet**

Zhenyuan Cai^1,2#^, Liuyang Wang^3#^, Xiaoying Song^4#^, Somnath Tagore^5#^, Xiangfeng Li^2#^, Huihua Wang^6^, Jiarui Chen^1,2^, Kexin Li^6,7^, Zeev Frenkel^7^, Dahai Gao^8^, Milana Frenkel-Morgenstern^5*^, Tongzuo Zhang^1,9*^, and Eviatar Nevo^7*^

^1^Key Laboratory of Adaptation and Evolution of Plateau Biota, Northwest Institute of Plateau Biology, Chinese Academy of Sciences, Xining 810008, China.^2^University of Chinese Academy of Sciences, No.19A Yuquan Road, Beijing 100049, China.^3^Department of Molecular Genetics and Microbiology, Duke University Medical Center, Durham, NC 27708, USA. ^4^School of Life Sciences, Zhengzhou University, Zhengzhou 450001, Henan, China. ^5^The Azrieli Faculty of Medicine, Bar-Ilan University, Safed 13195, Israel. ^6^Institute of Apicultural Research, Chinese Academy of Agricultural Sciences, Beijing, 100093, China.^7^The Institute of Evolution, University of Haifa, 199 Aba Khoushy Ave., Mount Carmel, Haifa 3498838, Israel.^8^Key Laboratory of Experimental Marine Biology, Institute of Oceanology, Chinese Academy of Sciences, Qingdao 266071, Shandong, China.^9^Qinghai Provincial Key Laboratory of Animal Ecological Genomics, Xining 810008, China.

^#^Authors contributed equally to this work.

^*^Correspondence to **Eviatar Nevo** (nevo@research.haifa.ac.il)

Institute of Evolution, University of Haifa

199 Aba Khoushy Ave., Mount Carmel, Haifa, 3498838, ISRAEL

Tel: (972) 4 8240448, Fax: (972) 4 8246554

or **Milana Frenkel-Morgenstern** (milana.morgenstern@biu.ac.il)

Faculty of Medicine, Bar-Ilan University

8 Henrietta Szold, Safed 13195, Israel

or **Tongzuo Zhang** (zhangtz@nwipb.cas.cn)

Northwest Institute of Plateau Biology, Chinese Academy of Sciences

23XinningRoad, Xining810008, Qinghai, China

Tel: (86) 9716105845, Fax: (86) 9716143282

**Short title:** Adaptive transcriptome of zokor in Tibet

**This PDF file includes:**

## Materials and Methods

## Supplementary Tables S1-S14

## Additional file 1-2 notes

## Supplementary Figures S1-S15

## Supplementary Methods

**Sample collection**

Wild plateau zokors (*M. baileyi*) (Fig.1a) were captured alive from three altitudes in eastern of Qinghai-Tibet Plateau in the middle of October, 2013 (Table S1). The first sample site is mountainous agricultural land at elevation of 2845m, which supply *M. baileyi* with adequate food (N=3) (Fig.1b). The second sample collecting site is shrubby grassland at altitude of 3,280m (N=3) (Fig.1c). The third collecting site is alpine meadow at altitude of 3,715m (N=3) with poor food sources, approaching the species upper distributional range in Tibet at 4,600 m (Fig.1d). Animals were sacrificed in the field by injecting Ketaset CIII (Fort Dodge, USA) at 5 mg/kg of body weight, approved by the ethical committee of Northwest Institute of Plateau Biology, Chinese Academy of Sciences. Brain tissues were preserved in liquid nitrogen immediately.

**RNA isolation, cDNA library construction and sequencing**

Total RNA was isolated from the whole brain of each individual using RNeasy mini Kit (Qiagen, Germany) according to the manual. The quantity and quality of the RNA were assessed by spectrophotometry (Nanodrop), gel electrophoresis and Agilent 2100 (Agilent Technologies), respectively, and only the samples with RIN value ≥7 were used for the downstream analysis. Messenger RNA with poly(A) tail was isolated from 10 ug total RNA using Sera-mag magnetic oligo (dT) beads (Illumina, San Diego, CA). The purified mRNA was fragmented into pieces from 100 to 400 bp with divalent cations by incubation at 94 ℃ for 5 min. The first strand synthesis was conducted using random primers. Double-strand cDNA was synthesized using the double-stranded cDNA synthesis kit (Invitrogen, CA). The cDNA was subjected to end-repair and phosphorylation, 3’ adenylation, adaptor ligation, size selection (200bp) and PCR amplification using Truseq RNA library preparation kit V2 (Illumina, San Diego, CA) following the manufacturer’s protocols. Each sample was prepared with a unique index. After quantification by Qubit® 2.0 fluorometer (Life Technologies, USA) and qPCR (LightCycler® 480 Instrument II, Greece), cluster generation was conducted on cBot (Illumina, San Diego, CA), and later 100bp pair end sequencing were conducted on HiSeq 2000 (Illumina, San Diego, CA).

**Transcriptome assembly**

Three individuals, all adult males, from each group and 3 altitudinal groups, ranging in weight from 265g to 399g (Table S1) were studied. Pair end libraries of the 9 samples were prepared and sequenced on Illumina HiSeq 2000 platform. All the reads were sorted by barcodes. Adaptor sequences were clipped and low quality reads were filtered out by Fast-Tool kit (1) and home-made transcript. These low-quality reads include reads with >10 nucleotides aligned to the adapter sequences, those of putative PCR duplicates, those with average base quality <15, those with >50% having a base quality score <10, and those with >5 % unidentified nucleotides (N). The generated clean reads used for following de novo assembly.

Base quality was checked and visualized by FASTQC (2). The clean reads from the 9 individuals were pooled separately for downstream de novo assembly with Trinity (3, 4). Each individual, three individuals at the same elevation and all the nine individuals were assembled separately. Contigs less than 200bp were removed from further analysis. The unigenes generated from each sample were taken into further assembly and redundancy removal by using TGICL (5) and Phrap (6) in order to acquire non-redundant unigenes as long as possible. The length distribution of all unigenes, blasted CDS, and predicted CDS were calculated.

**Functional annotations**

The redundant shorter unigenes were filtered and only the longest isotig left. Sequence homology searches were carried out using BLAST all programs against sequences in NCBI databases of nucleotide *(Nt*), BlastX against non-redundant (*Nr*), Swissprot, “KEGG (Kyoto Encyclopedia of Genes and Genomes)”, COG (Cluster of Orthologous Groups of proteins) databases with E-value<10^-6^. The unmapped unigenes were predicted by ESTscan (7). The length distribution of unigenes was estimated by homemade script. Blast2GO (8) were used for gene ontology (GO) analysis with an e-value of 1e-6. Gene pathway network was generated through KEGG analysis by using KEGG Automatic Annotation Server (KAAS) (http://www.genome.jp/tools/kaas/) with an e-value of 1e-10.

**Variant calling**

Single nucleotide polymorphism (SNP) was called by SOAPsnp (http://soap. genomics.org.cn/soapsnp.html). The contigs with less than 2× coverage were removed from downstream analysis. Pair end reads were mapped to the transcript consensus using SOAP. The generated files were reordered after index building with Picard tools (http://picard.sourceforge.net). Duplicates were masked and the generated files were indexed again. In order to minimize the mapping error, local realignment around INDELs were conducted again using Picard tools. Base Recalibration was conducted in case of systematic error modes. All the SNPs with coverage less than 10 and quality score less than 20 were removed from downstream analysis. SNPs with quality score lower than 20 and read depth less than 2 are filtered out. In order to assure that each SNP was real, the SNPs present in three replicates of the same altitude were summarized. This means that read depth for each SNP is at least 6 for each altitudinal population studied. SNPs shared by three altitudes, and SNPs unique to each altitude, were calculated by homemade script.

**SNP density, PCA, Population structure and NJ phylogenetic tree construction of the three altitudes**

SNP density was calculated using window slide method with window size of 500kb. SNP densities between low and high, middle and high altitude samples were compared and chi-square test was performed. The distribution of SNPs in CDS (coding DNA sequence), 5’UTR (untranslated region) and 3’UTR (untranslated region) were checked. Synonymous and non-synonymous mutations were estimated. Principle component analysis (PCA) was performed by R software with the SNPs called above and the figure is done by R software. Population structure was investigated on the *L, M,* and *H* populations based on the full maximum likelihood method and implemented in the program frappe v1.1 (9). This analysis was carried out on the whole dataset of SNPs. The probable number of ancestral populations was set to 2, 3, and 4, respectively, according to the ecological information and the number of individuals. A phylogenetic tree by neighbor-joining method use fneighbor software (http://bioinf.ibun.unal.edu.co/cgi-bin/emboss/help/fneighbor) according to the SNP site information. cDNA sequences of *EGLN1*for other 25 species and *EPAS1* for other 25 species were downloaded from NCBI (http://www.ncbi.nlm.nih.gov/), and alignment was conducted by Clustal X 2.0 (10). Highlight of the alignment was performed by BoxShade (11).

**Differential expression of transcripts among the three altitudes**

Differentially expressed genes (DEGs) of zokors from the three different elevation groups were calculated with NOIseq (12). GO enrichment was performed on the DEGs with using the DAVID (Database for Annotation, Visualization and Integrated Discovery) (13). DEGs, designated as Uniprot IDs, were submitted to the DAVID for functional enrichment analysis. Benjamini-corrected modified Fisher’s exact tests was used to examine the significance of the enrichment, and *P* values (i.e. EASE scores) smaller than 0.05 were considered as significant. RPKM (reads per kilobase of exon model per million mapped reads) (14) for each gene from each altitude sample were calculated. Differential expressed genes with fold change ≥2 and probability of 0.95 were enriched according to the pipeline developed by Yang (15). Heat maps were generated by clustering analysis using *ggplot2*package (16). Volcano plots were generated in R using *limma* package. The clusters of transcripts with common expression files were extracted by R and the number of clusters was set to nine (17). The expression level of each transcript was log2-transformed before plotting data points.

**Evolutionary analyses**

We calculated the *F*_ST_ (18), *G*_ST_ (19), and Tajima’s *D* (20) to capture the loci under natural selection. The *F*_ST_ and *G*_ST_ among pairwise groups and Tajima’s *D* for each group were calculated by the io::PopGen::PopStats package in BioPerl (21). Differentiation degree of transcripts was calculated using Locus-specific branch lengths (LSBL) (22). Following Shriver *et al*. 2004 (22), LSBL was calculated from single locus pairwise *F*_ST_ distances, where LSBL_(H)_ = [_(H-M)_*F*_ST_ + _(H-L)_*F*_ST_ − _(H-L)_*F*_ST_]/2. The differentiated genes and transcripts were subjected to perform functional enrichment of the candidate genes using, the ClueGO plugin from Cytoscape 3.2.1 (23) with, *Mus Musculus* as background species. *P* values less than 0.05 after Bonferroni correction for multiple testing were considered statistically significant.

**Validation of differentially expressed genes by qRT-PCR**

Nine significantly expressed genes, including 7 up- and 2 down-regulated unigenes, were selected for quantitative real-time RT-PCR (qRT-PCR) analysis to evaluate the expression patterns deduced from the sequencing data. Primers were designed based on contig sequences using Primer Premier 6 (24) (Table S8). cDNA was synthesized from the same total RNA samples used for RNA-seq by PrimeScript™ RT reagent Kit with gDNA Eraser (TaKaRa) according to manufacturer’s protocol. All the cDNA products were diluted to 90 ng/µl and utilized for the qRT-PCR. qPCR was performed with the SYBR® Premix Ex Taq™II (TaKaRa) in 384-well or 96-well plates on Applied BiosystemsViiA™ 7 Dx Real-Time PCR System. â-Actin was used as the reference gene for data normalization, and one sample from altitude 2846 meters (L) was used as the reference for relative fold changes. Fold changes from qRT-PCR were compared with the RNA-Seq expression analysis results. The correlation between the RNA-Seq and qRT-PCR results was analysed by calculating the coefficient of determination (R). Each sample had three technical replicates.

**Meta-analysis**

*Problem:*

The sampling of 3 individuals from each site is underpowered for the genetic diversity and selection analyses.

*Study selection:*

We made a randomization of the samples based on the SNP variation with respect to altitude.

*Summary measures:*

We used the Hedges' g as the summary measure for continuous data that is standardized to eliminate scale differences.

*Regression model, Normalization:*

We used the random meta-regression model to examine the impact of moderator variables on study effect size (Original sample) and Normalization, then re-meta-regression analysis.

## Supplementary Tables

**Table S**1 Sample sites and morphological information for all samples

| **Sample** | **Location(coordinates)** | | **Altitude (m)** | **Gender** | **Weight (g)** | **Size (cm)** | **Age** |
| --- | --- | --- | --- | --- | --- | --- | --- |
| L1 | E 101°47.478’ | N 37°05.577’ | 2843 | ♂ | 396 | 22.5 | Adult |
| L2 | E 101°47.455’ | N 37°05.568’ | 2848 | ♂ | 265 | 20 | Adult |
| L3 | E 101°47.403’ | N 37°05.589’ | 2848 | ♂ | 399 | 23 | Adult |
| M1 | E 101°45.544’ | N 37°11.853’ | 3271 | ♂ | 321 | 20.5 | Adult |
| M2 | E 101°45.541’ | N 37°11.878’ | 3279 | ♂ | 387 | 20 | Adult |
| M3 | E 101°45.572’ | N 37°11.910’ | 3296 | ♂ | 346 | 20 | Adult |
| H1 | E 101°25.330’ | N 36°21.586’ | 3715 | ♂ | 230 | 17.5 | Adult |
| H2 | E 101°25.295’ | N 36°21.630’ | 3720 | ♂ | 295 | 19.5 | Adult |
| H3 | E 101°25.306’ | N 36°21.542’ | 3708 | ♂ | 354 | 20 | Adult |

Note: L denotes low altitude, M denotes middle altitude, and H denotes high altitude

**Table S2** Environmental stresses at the three altitudes

| Altitude | Temperature | O_2_ | UV | Food |
| --- | --- | --- | --- | --- |
| 2,846 | 4.09 | 14.16% | 13.9324 | 3 |
| 3,282 | 1.48 | 13.70% | 13.9676 | 2 |
| 3,714 | -1.11 | 12.98% | 14 | 1 |

Note: Temperature at altitude of 2,846m is from the Xining Weather Bureau and others was estimated from the following website, so was the O_2_ (http://keisan.casio.com/exec/system/1224579725). UV was calculated according to the paper Blumthaler et al., 1997. Food is just defined as 1, 2, and 3, respectively.

**Table S3** Table 1 Summary of the reads generated for the nine zokors

| Samples | Total Raw Reads | Total Clean Reads | Total Clean Nucleotides (Nt) | Q20 percentage | N percentage | GC percentage |
| --- | --- | --- | --- | --- | --- | --- |
| L1 | 47,463,614 | 46,487,448 | 4,648,744,800 | 97.70% | 0.00% | 49.66% |
| L2 | 46,002,406 | 44,994,868 | 4,499,486,800 | 97.55% | 0.00% | 49.44% |
| L3 | 41,753,816 | 40,745,128 | 4,074,512,800 | 99.08% | 0.00% | 48.83% |
| M1 | 47,568,988 | 46,528,156 | 4,652,815,600 | 97.59% | 0.03% | 48.51% |
| M2 | 42,369,766 | 41,406,396 | 4,140,639,600 | 97.80% | 0.00% | 48.47% |
| M3 | 49,187,798 | 48,105,794 | 4,810,579,400 | 97.93% | 0.00% | 48.90% |
| H1 | 50,792,322 | 49,702,754 | 4,970,275,400 | 97.61% | 0.00% | 48.52% |
| H2 | 44,983,648 | 44,034,724 | 4,403,472,400 | 97.53% | 0.00% | 48.95% |
| H3 | 46,835,964 | 45,895,706 | 4,589,570,600 | 97.63% | 0.00% | 48.70% |

Note: Q20 denotes the probability of an incorrect base call in 100 bp sequencing read. N denotes the base pair is unknown.

**Table S4** Transcriptome assembly summary for each individual and combination of all samples

|  | Sample | Total Number | Total Length(Nt) | Mean Length(Nt) | N50 | Total Consensus Sequences | Distinct Clusters | Distinct Singletons |
| --- | --- | --- | --- | --- | --- | --- | --- | --- |
| Contig | L1 | 475,634 | 112,774,222 | 237 | 297 | - | - | - |
|  | L2 | 335,170 | 89,448,424 | 267 | 489 | - | - | - |
|  | L3 | 364,937 | 89,947,391 | 246 | 367 | - | - | - |
|  | M1 | 397,377 | 98,781,410 | 249 | 376 | - | - | - |
|  | M2 | 395,764 | 92,756,068 | 234 | 299 | - | - | - |
|  | M3 | 386,656 | 97,886,738 | 253 | 408 | - | - | - |
|  | H1 | 473,147 | 115,380,279 | 244 | 329 | - | - | - |
|  | H2 | 340,077 | 90,223,667 | 265 | 474 | - | - | - |
|  | H3 | 300,196 | 85,484,278 | 285 | 571 | - | - | - |
| Unigene | L1 | 220,917 | 140,854,510 | 638 | 1246 | 220,917 | 46,927 | 173,990 |
|  | L2 | 144,922 | 139,386,839 | 962 | 2452 | 144,922 | 36,545 | 108,377 |
|  | L3 | 144,973 | 124,868,884 | 861 | 2242 | 144,973 | 32,287 | 112,686 |
|  | M1 | 174,902 | 112,155,826 | 641 | 1247 | 174,902 | 35,483 | 139,419 |
|  | M2 | 159,075 | 93,448,803 | 587 | 1170 | 159,075 | 27,082 | 131,993 |
|  | M3 | 178,910 | 110,262,804 | 616 | 1108 | 178,910 | 36,834 | 142,076 |
|  | H1 | 219,147 | 139,114,621 | 635 | 1219 | 219,147 | 46,370 | 172,777 |
|  | H2 | 165,907 | 107,063,080 | 645 | 1184 | 165,907 | 36,025 | 129,882 |
|  | H3 | 161,962 | 108,638,187 | 671 | 1206 | 161,962 | 35,835 | 126,127 |
|  | All | 233,547 | 322,595,992 | 1381 | 2928 | 233,547 | 86,139 | 147,408 |

**Table S5** SNP number as in transition, transversion of the nine individuals from three elevations

| SNP Type | L1 | L2 | L3 | M1 | M2 | M3 | H1 | H2 | H3 |
| --- | --- | --- | --- | --- | --- | --- | --- | --- | --- |
| Transition | 13,160 | 11,456 | 9,800 | 11,700 | 8,882 | 12,559 | 13,964 | 19,419 | 19,528 |
| A->G | 6,657 | 5,762 | 4,923 | 5,894 | 4,403 | 6,290 | 6,998 | 9,765 | 9,796 |
| C->T | 6,503 | 5,694 | 4,877 | 5,806 | 4,479 | 6,269 | 6,966 | 9,654 | 9,732 |
| Transversion | 4,856 | 4,072 | 3,445 | 4,499 | 3,318 | 4,735 | 4,902 | 7,140 | 6,932 |
| A->C | 1,238 | 1,034 | 886 | 1,116 | 847 | 1,202 | 1,285 | 1,914 | 1,771 |
| A->T | 879 | 715 | 626 | 871 | 641 | 904 | 869 | 1,277 | 1,334 |
| C->G | 1,476 | 1,271 | 1,026 | 1,338 | 967 | 1,400 | 1,450 | 2,056 | 1,966 |
| G->T | 1,263 | 1,052 | 907 | 1,174 | 863 | 1,229 | 1,298 | 1,893 | 1,861 |
| Total | 18,016 | 15,528 | 13,245 | 16,199 | 12,200 | 17,294 | 18,866 | 26,559 | 26,460 |

**Table S6**. SNP density of the nine samples from the three altitudes

| Sample | N/Mbp | Mean | SD | Site pair comparison | *P* value |
| --- | --- | --- | --- | --- | --- |
| L1 | 48.61 | 45.07±2.45 | 4.24 | L&M | 0.455 |
| L2 | 45.82 |
| L3 | 40.77 |
| M1 | 44.79 | 42.29±5.62 | 9.74 | L&H | 0.005 |
| M2 | 37.39 |
| M3 | 44.69 |
| H1 | 89.36 | 78.48±2.29 | 3.97 | M&H | 0.004 |
| H2 | 70.58 |
| H3 | 75.49 |

**Table S7. Other parameters associated with altitude**

|  | *Pn* | maf | *H_o_* | *H_e_* | Π  (e-6) | θ  (e-6) |
| --- | --- | --- | --- | --- | --- | --- |
| H | 41516/83312 | 0.094 | 0.13 | 0.19 | 5.84 | 5.56 |
| M | 24733/83312 | 0.084 | 0.1 | 0.11 | 3.39 | 3.26 |
| L | 29382/83312 | 0.141 | 0.11 | 0.13 | 3.9 | 3.88 |

**Table S8** List of selected unigenes examined for qRT-PCR validation

| Unigene ID | Primers | |
| --- | --- | --- |
|  | Forward | Reverse |
| β-Actin | 5'-CTAAGGCCAACCGTGAAAAGAT-3' | 5'-GACCAGAGGCATACAGGGACA-3' |
| CL1550.Contig4 | 5'-TGTTGCTTCCGTAACCTGCCTA-3' | 5'-GCTTCGTTGAGATCCCAATTCC-3' |
| CL6395.Contig3 | 5'-CCCAGCACTGTATGTTTCCCAC-3' | 5'-CCCTTACGATACACCATCTCATTTC-3' |
| CL13954.Contig3 | 5'-CCCTGCCACCTCAGACAGAATAG-3' | 5'-ATTAAATCACATGCCAGAATCCCT-3' |
| Unigene27747 | 5'-ATGGAGGGAAGGAGGACATAACA-3' | 5'-CCTAAGCTCACAGCACCTGACTATG-3' |
| Unigene69559 | 5'-CGTGGCTGCGTAGTCGTTG-3' | 5'-AGGCTCAGGCAGGTGAACAG-3' |
| Unigene127082 | 5'-TCTTTCGTGTTTCCTGTGATTGAG-3' | 5'-ACTTCCCTGGCTCTTAGGGTTATG-3' |
| Unigene130644 | 5'-AGCCCAGAATGACGGAGACAA-3' | 5'-ATTGGATGCTGTGAACCTGCTAT-3' |
| CL17844.Contig1 | 5'-GATGGTTGATCTGTACTGGCGTAAC-3' | 5'-AGCCTGAAGGAGGGACTGCA-3' |
| Unigene92646 | 5'-TAAGCAGAGGGAAGAGTTAGCCA-3' | 5'-CCTCACTGTTTCAGCCCTCATC-3' |

Table S9 List of selected genes in Spalax for hypoxia tolerance

| GeneID | Symbol |
| --- | --- |
| 29560 | Hif1a |
| 83785 | Vegfa |
| 24835 | Tnf |
| 29452 | Epas1 |
| 24451 | Hmox1 |
| 24599 | Nos2 |
| 24323 | Edn1 |
| 24225 | Bdnf |
| 24600 | Nos3 |
| 59086 | Tgfb1 |
| 29527 | Ptgs2 |
| 24494 | Il1b |
| 24482 | Igf1 |
| 24185 | Akt1 |
| 24224 | Bcl2 |
| 24842 | Tp53 |
| 29260 | Tlr4 |
| 24598 | Nos1 |
| 81687 | Mmp9 |
| 24770 | Ccl2 |
| 50689 | Mapk3 |
| 24392 | Gja1 |
| 81686 | Mmp2 |
| 24330 | Egr1 |
| 25608 | Lep |
| 81649 | Mapk14 |
| 56718 | Mtor |
| 24498 | Il6 |
| 116590 | Mapk1 |
| 24179 | Agt |
| 81736 | Nfkb1 |
| 25402 | Casp3 |
| 25464 | Icam1 |
| 83810 | Trpv1 |
| 308913 | Egln1 |
| 314322 | Fos |

Table S10 List of selected genes in Spalax for hypercapnia tolerance

| GeneID | Symbol |
| --- | --- |
| 24451 | Hmox1 |
| 29560 | Hif1a |
| 29260 | Tlr4 |
| 81736 | Nfkb1 |
| 25085 | Th |
| 24408 | Grin1 |
| 116554 | Mapk8 |
| 24473 | Htr1a |
| 25601 | Oprm1 |
| 24335 | Epo |
| 114115 | P2rx2 |
| 29180 | Uts2 |
| 57305 | Uts2r |
| 54231 | Car2 |
| 361084 | Lmo7 |

**Table S11 List of selected genes in Spalax for ATP-pathway energetics**

| GeneID | Symbol |
| --- | --- |
| 29665 | P2rx7 |
| 24255 | Cftr |
| 25303 | Abcc2 |
| 81739 | P2rx3 |
| 29659 | P2rx4 |
| 114115 | P2rx2 |
| 24646 | Abcb1b |
| 170913 | Abcb1a |
| 25265 | P2ry1 |
| 25559 | Abcc8 |
| 312382 | Abcg2 |
| 171374 | Atp5b |
| 64519 | Entpd1 |
| 29718 | Kcnj10 |
| 25472 | Kcnj8 |
| 25560 | Abcc9 |
| 24565 | Abcc1 |
| 29597 | P2ry2 |
| 24225 | Bdnf |
| 24494 | Il1b |
| 25505 | P2rx1 |
| 24835 | Tnf |
| 59086 | Tgfb1 |
| 29527 | Ptgs2 |
| 24521 | Kcnj1 |
| 85333 | Slc25a4 |
| 313210 | Abca1 |
| 81649 | Mapk14 |
| 140668 | Abcc3 |
| 361436 | Afg3l1 |
| 691448 | Slc25a54 |
| 361875 | Atp6ap1l |

Table S12 List of selected genes in Spalax for temperature tolerance

| GeneID | Symbol |
| --- | --- |
| 83810 | Trpv1 |
| 24225 | Bdnf |
| 29527 | Ptgs2 |
| 24600 | Nos3 |
| 24185 | Akt1 |
| 81649 | Mapk14 |
| 25325 | Il10 |
| 24318 | Drd2 |
| 25125 | Stat3 |
| 56718 | Mtor |
| 24316 | Drd1 |
| 29290 | Adora1 |
| 29595 | Htr2a |
| 25723 | Hcrt |
| 24596 | Ngfr |
| 297376 | Htra2 |
| 24221 | Avp |
| 24241 | Calca |
| 59109 | Ntrk1 |
| 25601 | Oprm1 |
| 24473 | Htr1a |
| 25302 | Chrna7 |
| 24925 | Adrb1 |
| 312896 | Trpa1 |
| 266759 | Hspa4 |
| 81638 | Agtr1b |
| 24404 | Gpx1 |
| 309945 | Arrdc3 |
| 287822 | Hid1 |
| 688699 | Prdm12 |
| 1E+08 | Acot11 |

Table S13: Meta-analysis Method: 27 validity studies, N = 9

| **Study** | **Observed Validity Coefficient** |
| --- | --- |
| **1** | 0.11 |
| **2** | 0.22 |
| **3** | 0.45* |
| **4** | 0.23 |
| **5** | 0.18 |
| **6** | 0.12 |
| **7** | 0.21 |
| **8** | 0.11 |
| **9** | 0.23 |
| **10** | 0.34* |
| **11** | 0.27* |
| **12** | 0.32* |
| **13** | 0.21 |
| **14** | 0.12 |
| **15** | 0.19 |
| **16** | 0.22 |
| **17** | 0.17 |
| **18** | 0.41* |
| **19** | 0.22 |
| **20** | 0.12 |
| **21** | 0.47* |
| **22** | 0.42* |
| **23** | 0.31* |
| **24** | 0.21 |
| **25** | 0.18 |
| **26** | 0.28* |
| **27** | 0.29* |

*** p < 0.05 (two-tailed)**

Table S14: 95% confidence interval for correlations from Table S14, N = 9

| **Study** | **Observed Validity Coefficient** | **95% Confidence Interval: Lower** | **95% Confidence Interval: Upper** |
| --- | --- | --- | --- |
| **1** | 0.22 | -0.01 | 0.45 |
| **2** | 0.44* | 0.2 | 0.56 |
| **3** | 0.49* | 0.23 | 0.6 |
| **4** | 0.21 | -0.02 | 0.44 |
| **5** | 0.22 | -0.01 | 0.45 |
| **6** | 0.18 | -0.01 | 0.4 |
| **7** | 0.27* | 0.08 | 0.48 |
| **8** | 0.19 | -0.01 | 0.39 |
| **9** | 0.28* | 0.09 | 0.46 |
| **10** | 0.44* | 0.2 | 0.56 |
| **11** | 0.47* | 0.24 | 0.59 |
| **12** | 0.42* | 0.21 | 0.54 |
| **13** | 0.31* | 0.1 | 0.42 |
| **14** | 0.19 | -0.01 | 0.38 |
| **15** | 0.29* | 0.09 | 0.45 |
| **16** | 0.32* | 0.1 | 0.43 |
| **17** | 0.19 | -0.01 | 0.38 |
| **18** | 0.47* | 0.24 | 0.59 |
| **19** | 0.29* | 0.09 | 0.45 |
| **20** | 0.19 | -0.01 | 0.38 |
| **21** | 0.49* | 0.23 | 0.6 |
| **22** | 0.48* | 0.22 | 0.58 |
| **23** | 0.38* | 0.19 | 0.53 |
| **24** | 0.28* | 0.12 | 0.43 |
| **25** | 0.21 | -0.02 | 0.45 |
| **26** | 0.38* | 0.19 | 0.53 |
| **27** | 0.39* | 0.2 | 0.57 |

## Additional file notes

**Additional file 1:** Dataset of *F*_ST_ outlier genetic analysis.

**Additional file 2:** Dataset of *G*_ST_ outlier genetic analysis.

## Supplementary Figures


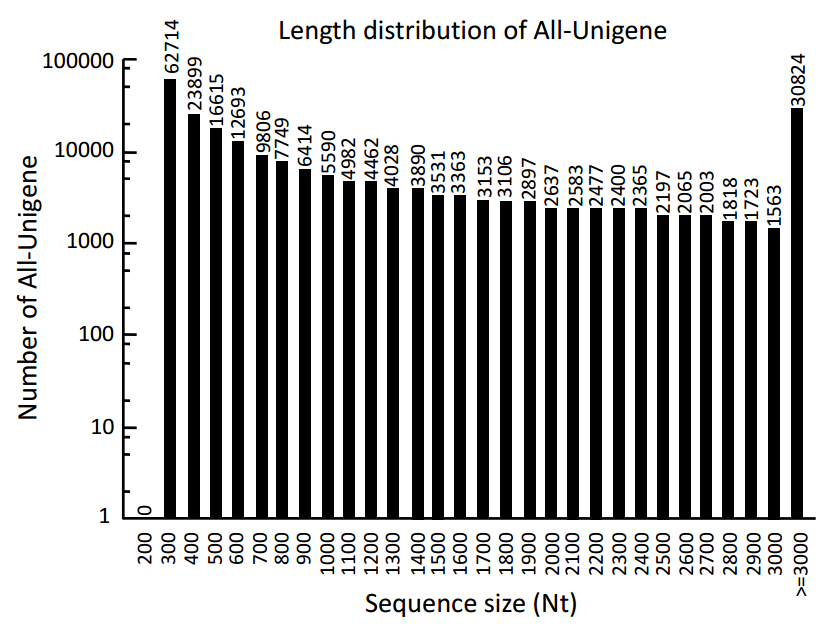


**Fig. S1.** The length distribution of all unigenes in nine individuals examined of *M. baileyi*.


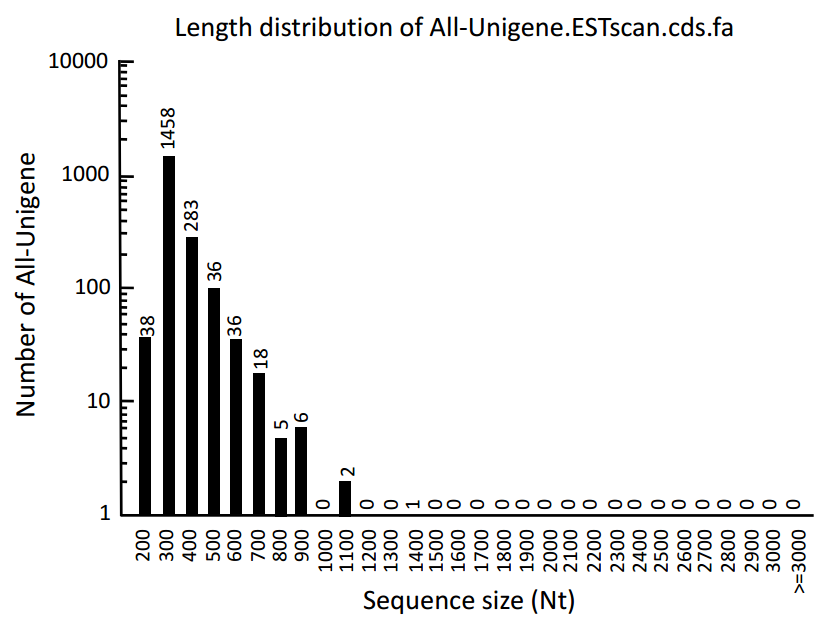


**Fig. S2.** The length distribution of CDS predicted from the unigenes left after annotation.


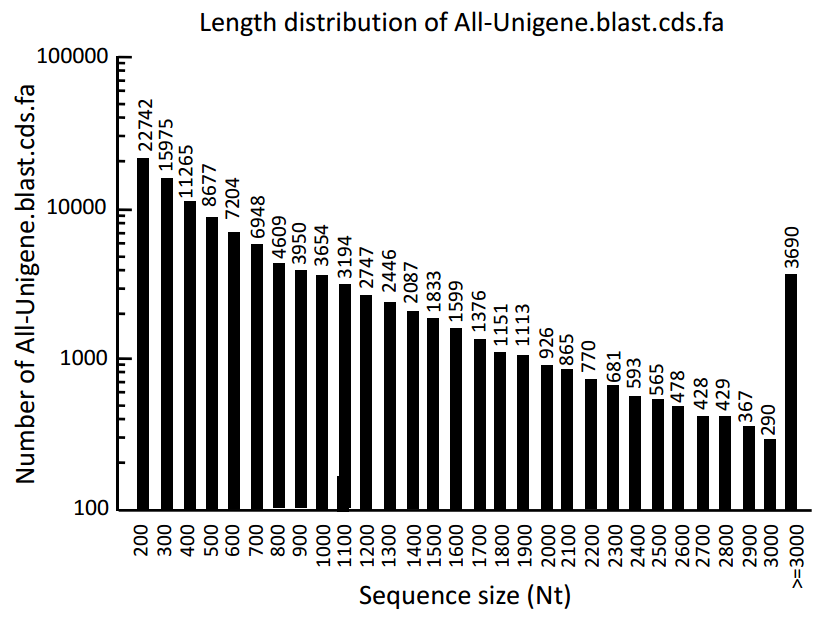


**Fig. S3.** The length distribution of CDS scanned from all the unigenes.

**
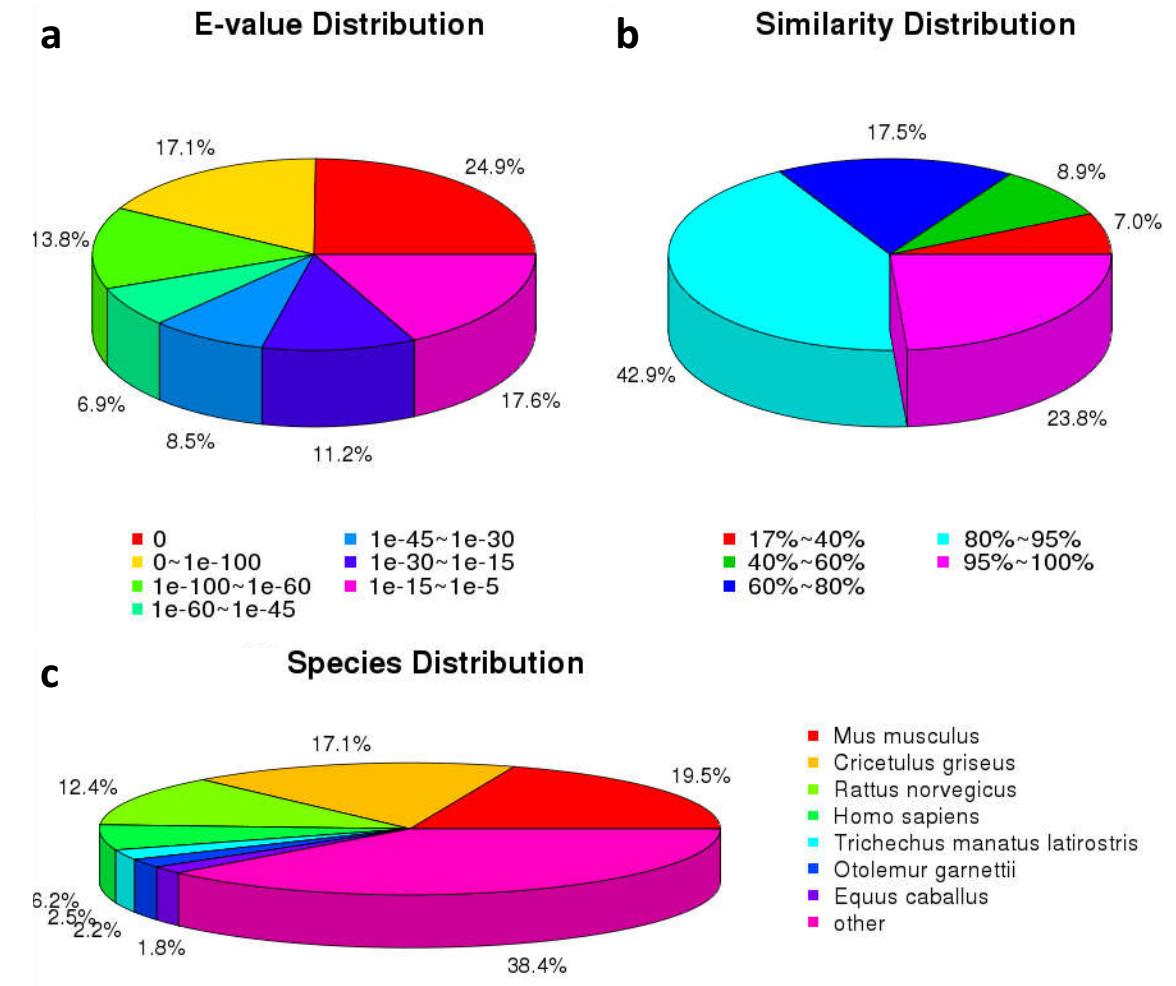
**

**Fig. S4** Figure of Nr classification. (a) E-value distribution, (b) Identity distribution, (c) Species distribution.


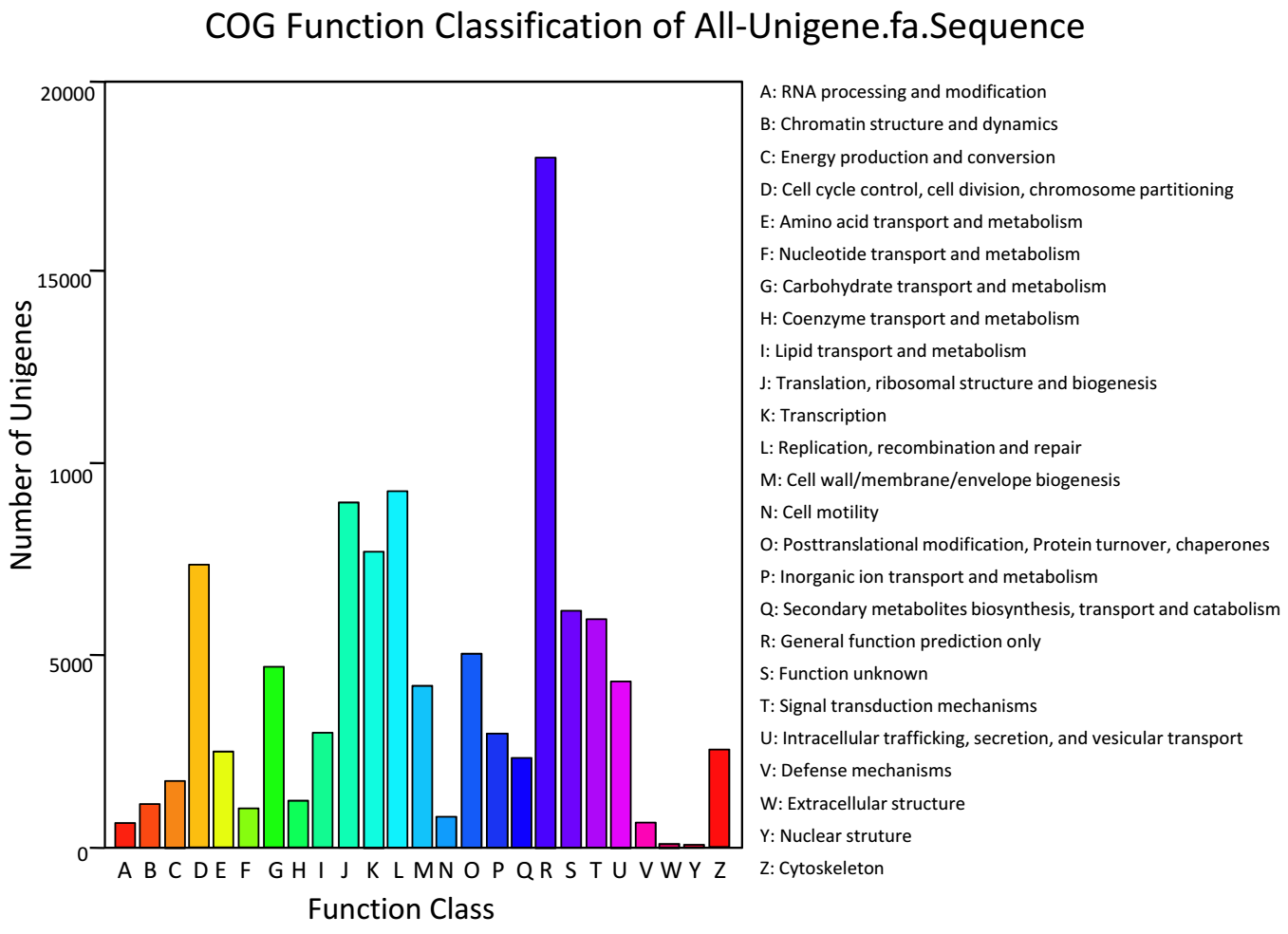


**Fig. S5** Histogram of clusters of orthologous groups (COG) classification. All unigenes were assigned to 25 specific COG categories. The x-axis denotes 25 groups of COG. The y-axis denotes the number of unigenes 14 annotated to each COG category.

**
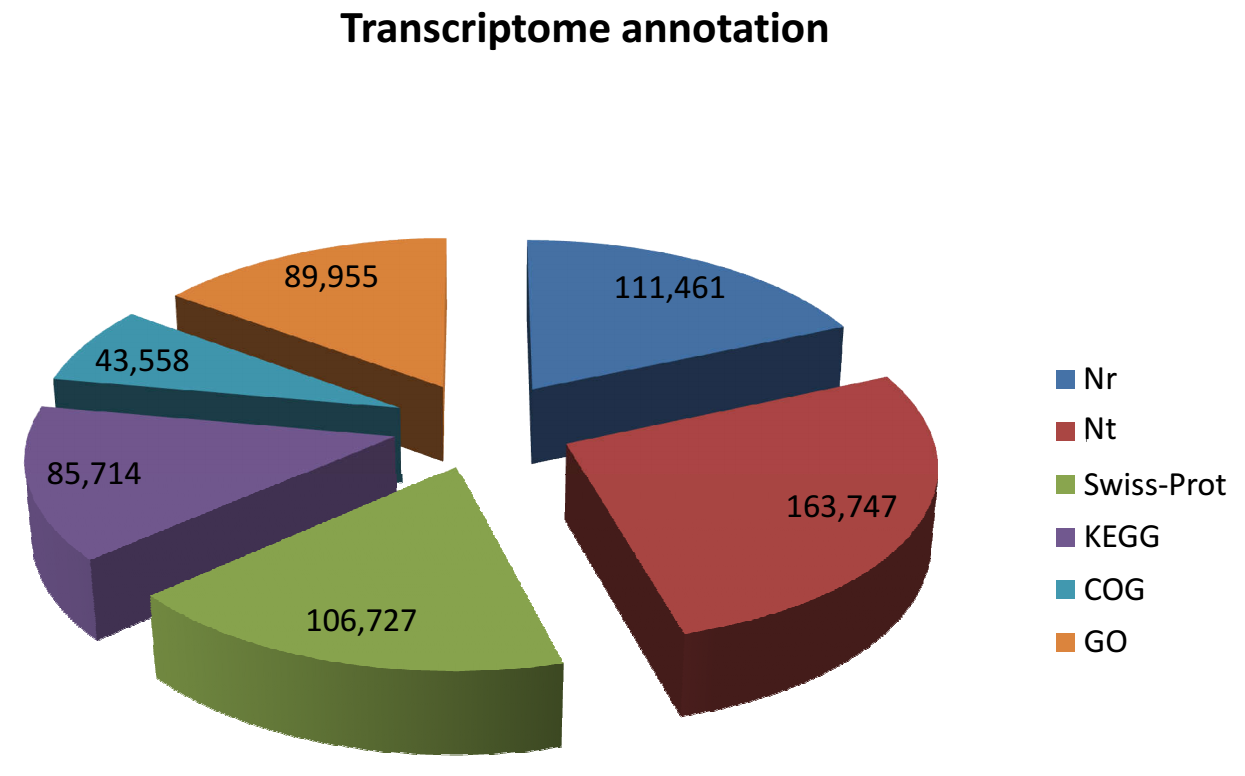
**

**Fig. S6** Number of transcripts annotated to each database. These databases include Non-redundant (Nr), Nucleotide database (Nt), Swiss-prot, “KEGG (Kyoto Encyclopedia of Genes and Genomes)”, COG (Cluster of Orthologous Groups of proteins) databases.

**
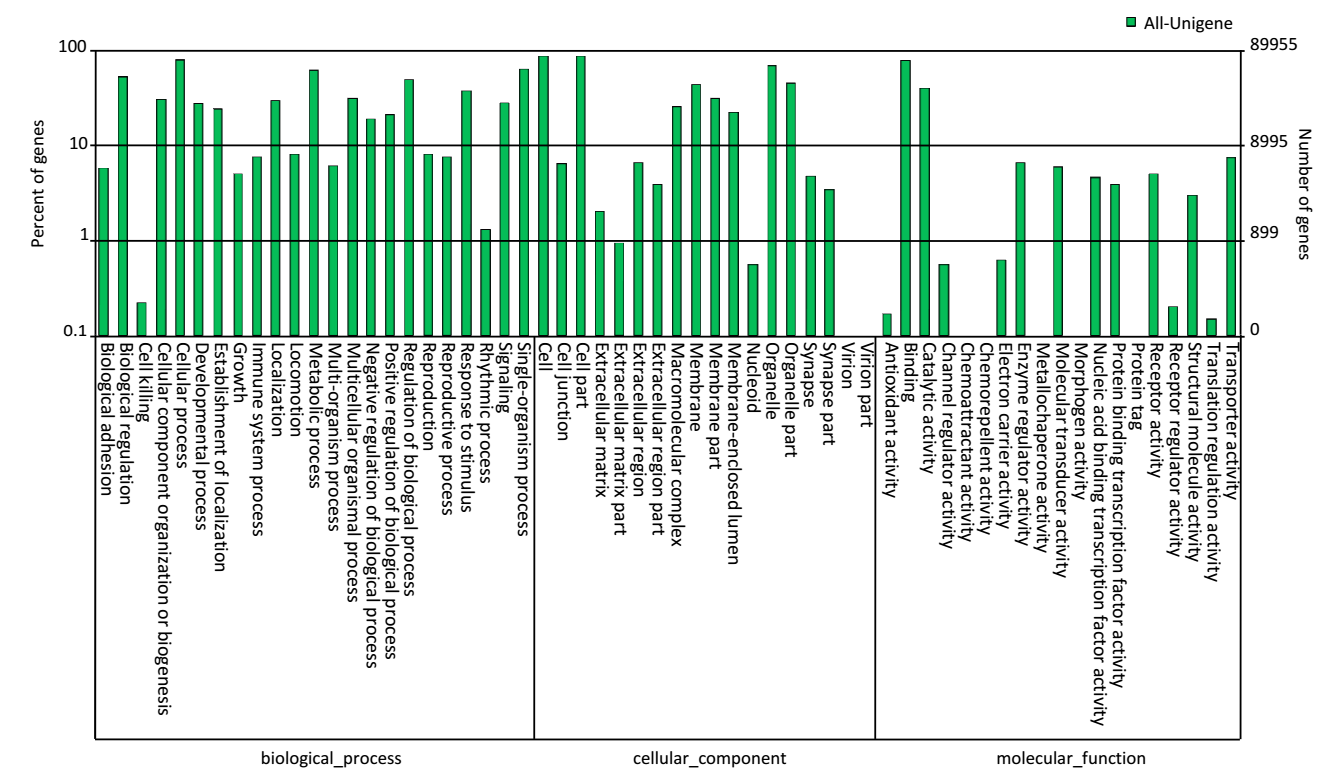
**

**Fig. S7** Histogram of gene ontology (GO) classification. The GO terms were classified into three main categories: biological process, cellular component and molecular function. The lefty-axis denotes the percentage of a certain category of genes in the main category. The righty-axis denotes the number of genes in a category.

**
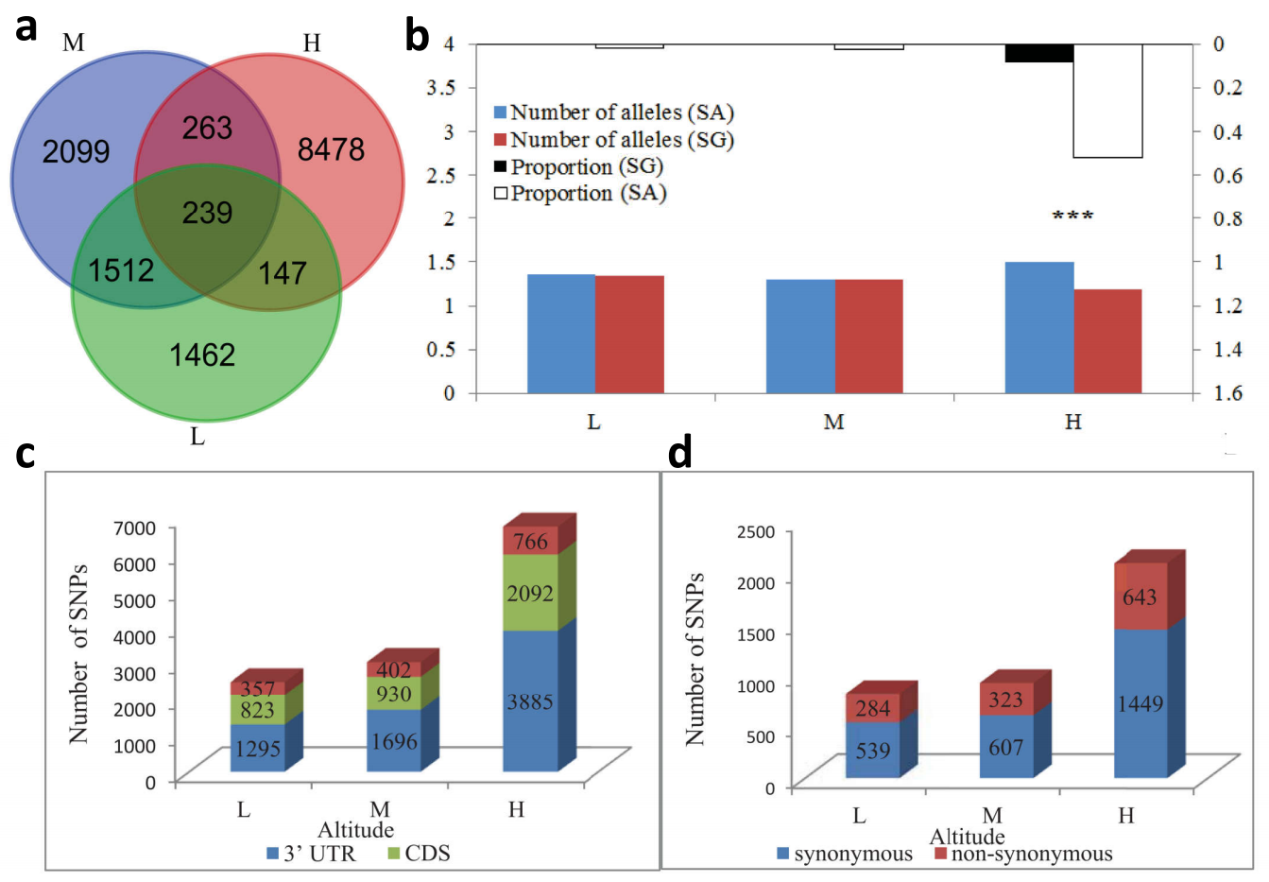
**

**Fig. S8** SNP comparisons of three altitudinal populations. (a) Venn figure of shared SNPs of different elevations and SNPs unique to each altitude. L represents SNPs discovered in low-altitude population (2,846 m); M represents SNPs discovered in middle-altitude population (3,282 m); H represents SNPs discovered in high-altitude population (3,714 m); the overlapping areas mean SNPs shared by different populations. The biggest jump in unique SNPs is in the 432 m from M to H. (b) The difference in allele number and proportion between gene coding area and all SNP datasets. (c) SNP distribution in 3'UTR, 5'UTR, and CDS at 3 altitudes. (d) Synonymous and non-synonymous SNPs at the 3 altitudes. Note the dramatic genetic change at the highest altitudinal population approaching the upper species border.

**Fig. S9.** Expression level of *Myospalax* transcripts from three altitudinal populations studied in Tibet. (a) Number of up- and down-regulated genes across the three sample sites (b) Gene expression pattern of all the unigenes for nine clusters. The horizontal axis denotes each altitude, L, low altitude (2,846m), M, middle altitude (3,282m) and H, high altitude (3,714m). The vertical axis indicates the log_2_ fold change calculated between every two altitudes. Note the dramatic difference between L vs M+L.

**Fig. S10.** Pairwise comparison show distinct number of differentially expressed genes among three altitudinal populations. (a), (b), and (c) represent the hierarchical clustering analysis of DEGs between animals from L-vs.-M, L-vs.-H and M-vs.-H pairs, respectively. L1, L2, and L3; M1, M2, and M3 and H1, H2, and H3 on the Y-axis represent animals from low, middle, and high-altitude populations, respectively. X-axis denotes each DEG. (d), (e), and (f) were volcano plots of DEGs for L-vs.-M, L-vs.-H and M-vs.-H pairs, respectively. The X-axis represents the log_2_(fold-change) between every two altitudes and the Y-axis represents the –log_10_(*P*) value for statistical significance.

**
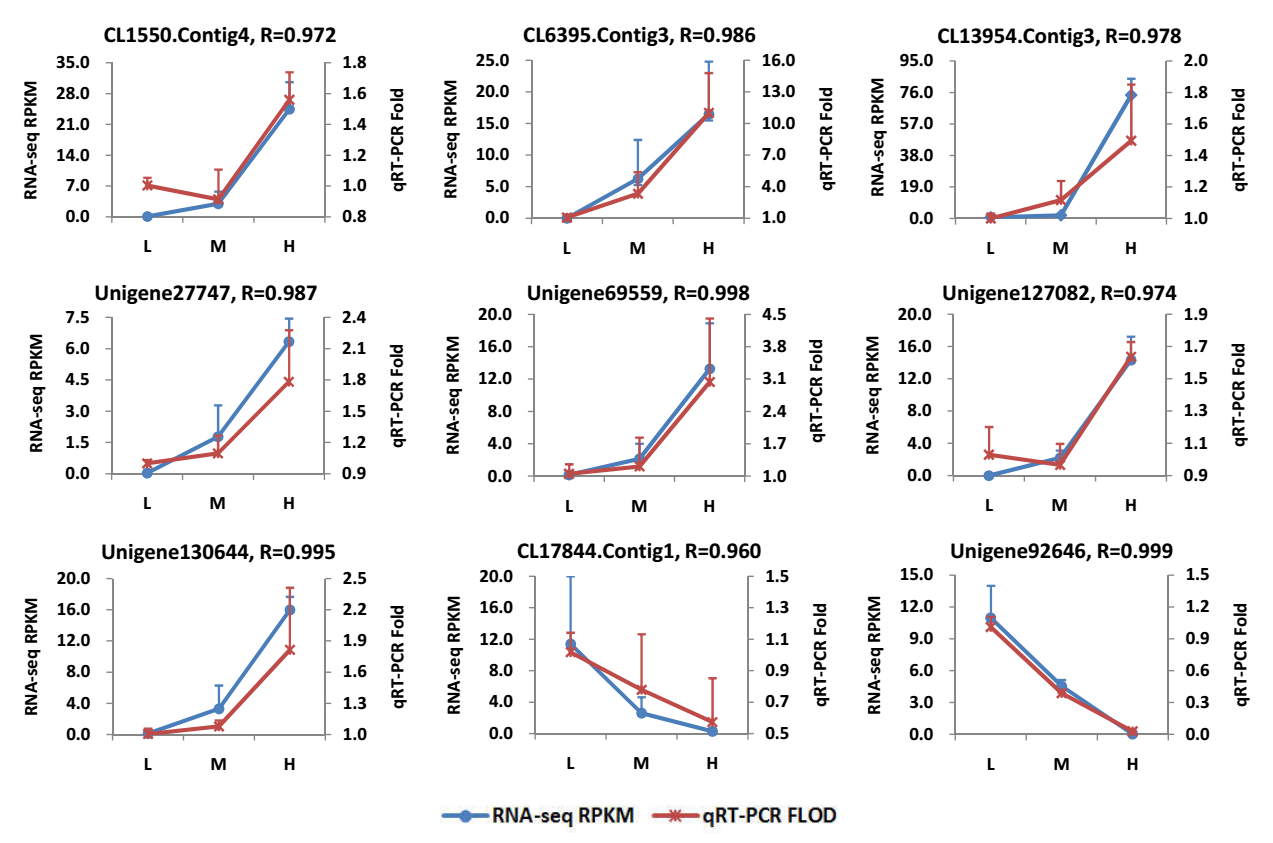
Fig. S11.** qRT-PCR validation results for nine selected unigenes. The consistent trend of up- or down-regulation in 9 unigenes between qRT-PCR and RNA-Seq data confirmed the reliability of differential expression analysis performed in this study. Unigene IDs are available in Table S8. R is the coefficient of determination, which means the correlation between the RNA-Seq and qRT-PCR results. L represents low altitudinal population (2,846m), M represents middle altitudinal population (3,282m), H represents high altitudinal population (3,714m). RPKM is abbreviation of Reads per kilobase of exon model per million mapped reads. Fold changes are relative to one sample from low altitude 2,846 meters (L) values and normalized by changes in β-Actin values. Error bars reflect standard error of the mean. X-axis denotes the three altitudinal populations: Low, Middle, and High; The left Y-axis denotes the RPKM from transcriptome and the right Y-axis denotes the difference by RT-PCR. Note the dramatic difference between L and (M+H). To evaluate the reliability of RNA-seq, 7 up-regulated and 2 down-regulated unigenes were quantified by qRT-PCR analysis between different altitudinal pairs. This showed conformity to the transcriptome analyses.

**
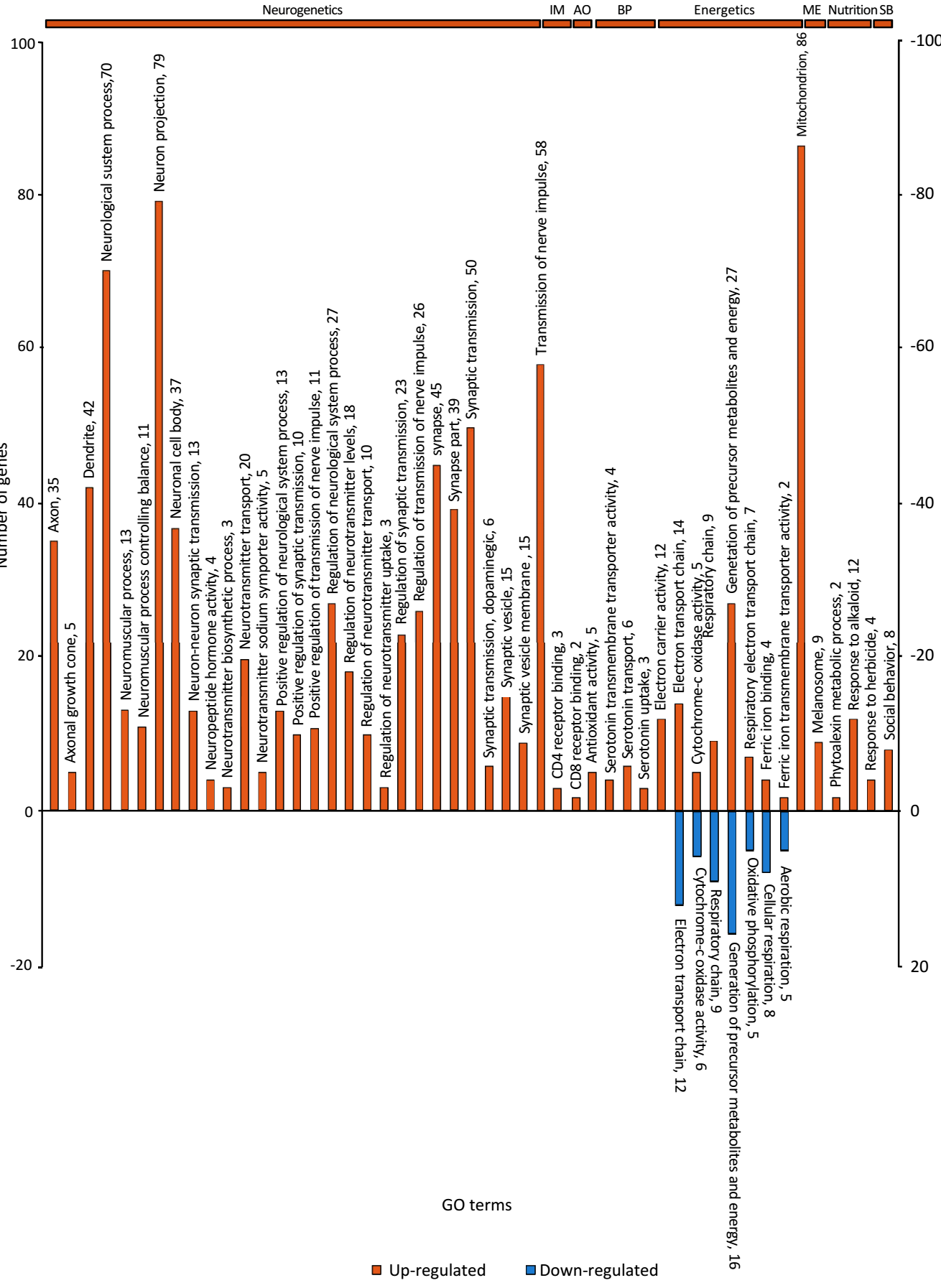
Fig. S12.** Gene ontology (GO) of up- and down-regulated zokor genes comparing for L and H populations.


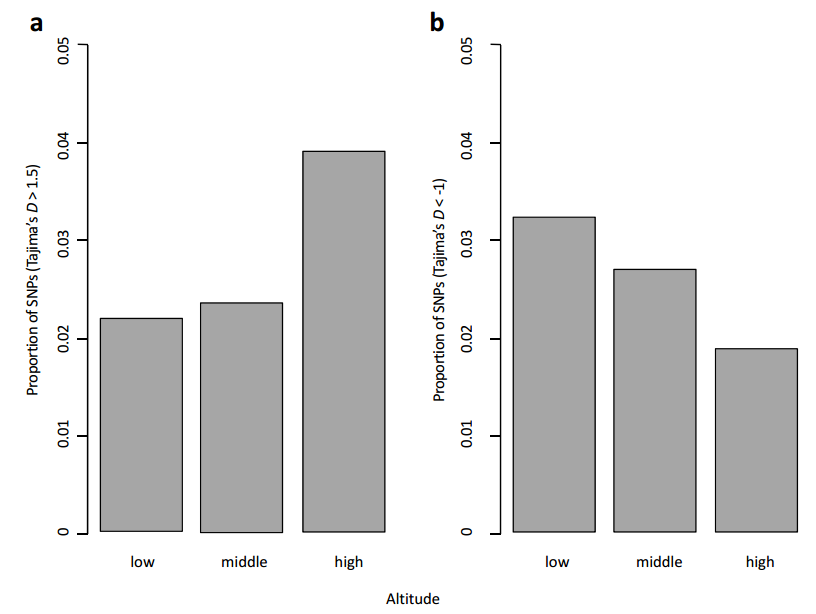


**Fig. S13** Proportion of SNPs with extreme Tajima’s *D* values indicates different population differentiation by natural selection of the three altitudinal populations, sharply divergent ecologically and genetically.

**Fig. S14** Amino acid substitution of *EGLN1* and *EPAS1* genes unique to *Myospalax* in the Tibet plateau. (a) Mutation unique to *M. bailey* in *EGLN1* gene across 25 species. (b) and (c) are mutations unique to *M. bailey* in *EPAS1* gene across 25 species. Myo denotes *M. bailey*. Note these unique mutations of *M. bailey* adapted to Tibetan stresses in comparison with non-Tibetan species. Heterocephalus: *Heterocephalus glaber*, Octodon: *Octodon degus*, NMR: *Heterocephalus glaber*, Microtus: *Microtus ochrogaster*, Peromyscus: *Peromyscus maniculatus*, RAT: *Rattus norvegicus*, BMR: *Spalax galili*, Cricetulus: *Cricetulus griseus*, Mesocricetus: *Mesocricetus auratus*, Cavia: *Cavia* *aperea*, Ovis: *Ovis aries*, Bos: *Bos mutus*, Felis: *Felis catus*, Bos-taurus: *Bos taurus*, Sus: *Sus scrofa*, Tupaia: *Tupaia chinensis*, Canis: *Canis lupus familiaris*, Chlorocebus: *Chlorocebus sabaeus*, HUM: *Homo sapiens*, mulatta: *Macaca-mulatta*, fascicularis: *Macaca fascicularis*, Oryctolagus: *Oryctolagus cuniculus*, Condylura: *Condylura cristata*, Chrysochloris: *Chrysochloris asiatica*, Myo: *Myospalax baileyi*, Mus: *Mus musculus*, Callithrix: *Callithrix* *jacchus*, Chinchilla: *Chinchilla lanigera*, Tursiops: *Tursiops truncates*.


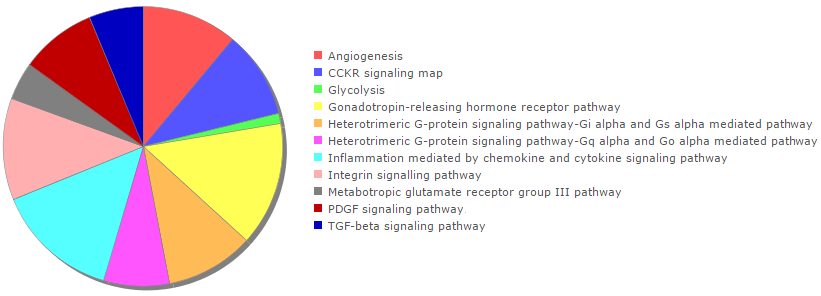


**Fig. S15** Annotation results of *Myospalax* genes reveal associations with various molecular pathways like cytokine signaling, angiogenesis, CCKR signaling, integrin signaling.

## References

1. Gordon A, Hannon GJ (2010) Fastx-toolkit. FASTQ/A short-reads preprocessing tools *( http://hannonlab.cshl.edu/fastx_toolkit)*.

2. Andrews S (2010) FastQC: A quality control tool for high throughput sequence data. *(http://www.bioinformatics.babraham.ac.uk/projects/fastqc/)*.

3. Grabherr MG, et al. (2011) Full-length transcriptome assembly from RNA-Seq data without a reference genome. *Nat Biotechnol* 29(7):644-652.

4. Grabherr MG, et al. (2013) Trinity: reconstructing a full-length transcriptome without a genome from RNA-Seq data. *Nat Biotechnol* 29(7):644-652.

5. Pertea G, et al. (2003) TIGR Gene Indices clustering tools (TGICL): a software system for fast clustering of large EST datasets. *Bioinformatics* 19(5):651-652.

6. Bastide M, McCombie WR (2007) Assembling genomic DNA sequences with PHRAP. *Current Protocols in Bioinformatics* Chapter 11: Unit11.4-Unit11.4.

7. Iseli C, Jongeneel CV, Bucher P (1999) ESTScan: a program for detecting, evaluating, and reconstructing potential coding regions in EST sequences. *ISMB* PP:138-148.

8. Conesa A, et al. (2005) Blast2GO: a universal tool for annotation, visualization and analysis in functional genomics research. *Bioinformatics* 21(18):3674-3676.

9. Tang H, Peng J, Wang P, Risch NJ (2005) Estimation of individual admixture: analytical and study design considerations. *Genet Epidemiol* 28(4):289-301.

10. Larkin MA, et al. (2007) Clustal W and Clustal X version 2.0. *Bioinformatics* 23(21):2947-2948.

11. Hofmann K, Baron M (1996) Boxshade 3.21. *Pretty printing and shading of multipe-alignment files. Kay Hofmann ISREC Bioinformatics Group, Lausanne, Switzerland*.

12. Tarazona S, García F, Ferrer A, Dopazo J, Conesa A (2012) NOIseq: a RNA-seq differential expression method robust for sequencing depth biases. *EMBnet. Journal* 17(B):18-19.

13. Glynn Dennis Jr, et al. (2003) DAVID: Database for Annotation, visualization, and Integrated Discovery. *Genome Biol* 4(5):3.

14. Mortazavi A, Williams BA, McCue K, Schaeffer L, Wold B (2008) Mapping and quantifying mammalian transcriptomes by RNA-Seq. *Nat Methods* 5(7):621-628.

15. Chen S, et al. (2010) De novo analysis of transcriptome dynamics in the migratory locust during the development of phase traits. *PloS ONE* 5(12):e15633.

16. Wickham H. (2009) *ggplot2: Elegant Graphics for Data Analysis*. (Springer, New York, USA).

17. Haas BJ, et al. (2013) De novo transcript sequence reconstruction from RNA-seq using the trinity platform for reference generation and analysis. *Nat Proto* 8(8):1494-1512.

18. Weir BS, Cockerham CC. (1984) Estimating F-Statistics, for the Analysis of Population Structure. *Evolution* 38(6):1358-1370.

19. Nei M, Chesser RK. (1983) Estimation of fixation indices and gene diversities. *Ann Hum Genet* 47(JUL):253-259.

20. Tajima F. (1983) Evolutionary relationship of DNA sequences in finite populations. *Genetics* 105(2):437-460.

21. Stajich JE, et al. (2002) The bioperl toolkit: perl modules for the life sciences. *Genome Res* 12(10):1611-1618.

22. Shriver MD, et al. (2004) The genomic distribution of population substructure in four populations using 8,525 autosomal SNPs. *Hum Genomics* 1(4):274-286.

23. Bindea G, et al. (2009) Cluego: a cytoscape plug-in to decipher functionally grouped gene ontology and pathway annotation networks. *Bioinformatics* 25(8):1091-1093.

24. Lalitha S. (2000) Primer premier 5. *Biotech Software & Internet Report*. 1(6):270-272.
